# Epigenetic modulation elicits an NK cell-mediated immune response in urothelial carcinoma

**DOI:** 10.1186/s10020-025-01264-9

**Published:** 2025-06-24

**Authors:** Himani Kumari, Ciao-Ni Chen, Hsin-An Shih, Chih-Chieh Yeh, Tsung-Yu Tseng, Hsing-Fen Tsai, Jie Ting Low, Chin Pui Chan, Guan-Ling Lin, Wan-Hong Huang, Chao-Ling Yao, Steven Lin, Cheng-Huang Shen, Michael W. Y. Chan

**Affiliations:** 1 Department of Biomedical Sciences, National Chung Cheng University, Min-Hsiung, Chia-Yi Taiwan; 2 Epigenomics and Human Diseases Research Center, National Chung Cheng University, Min-Hsiung, Chia-Yi Taiwan; 3 Center for Innovative Research on Aging Society (CIRAS), National Chung Cheng University, Min-Hsiung, Chia-Yi Taiwan; 4 Institute of Biological Chemistry, Academia Sinica, Taipei, Taiwan; 5 Department of Chemical Engineering, National Cheng Kung University, Tainan, Taiwan; 6 Department of Obstetrics and Gynecology, National Cheng Kung University Hospital, Tainan City, Taiwan; 7 Department of Urology, Ditmanson Medical Foundation Chiayi Christian Hospital, Chiayi, Taiwan; 8 Research Center for Precision Environmental Medicine, Kaohsiung Medical University, Kaohsiung, Taiwan; 9 Present address: Department of Molecular Biology and Human Genetics, Tzu Chi University, Hualien, Taiwan

**Keywords:** Urothelial carcinoma, Innate immunity, NKG2DL, ULBP2, CCL3, CPH, NK cells

## Abstract

Urothelial carcinoma (UC), the second most prevalent cancer of the urothelial system, has demonstrated a well-developed ability to evade natural killer (NK) cell-mediated killing via various pathways, highlighting the need for innovative therapeutic approaches. Our study examines the role of cyproheptadine (CPH) and entinostat (ENT) as epigenetic modifiers in enhancing NK cell-mediated cytotoxicity against UC. Through our in vitro experiments, we observed that pre-treatment with CPH or ENT significantly increased NK cell-mediated elimination of UC cells, as demonstrated in NK cytotoxicity assay wherein UC cells were co-cultured with both NK-92 and human primary NK cells. This enhancement is attributed to the epigenetic activation of NKG2D ligands, notably through increased H3K27ac enrichment and reduced enrichment of H3K27me3 at the ULBP2 promoter region in UC cells. Additionally, ectopic expression of ULBP2 in UC cells further augmented their susceptibility to NK cell-mediated killing. Importantly, our syngeneic mouse model using MB49 tumor cells, showed that CPH or ENT treatments significantly reduced tumor growth via promoting NK cell infiltration into the tumor microenvironment. This increased infiltration is likely due to elevated levels of the NK-recruiting chemokine CCL3 in drug-treated UC cells. Collectively, these results underscore the potential of CPH and ENT to suppress tumor growth and enhance immune surveillance by modulating epigenetic pathways that boost NK cell activity, offering promising insights into new strategies for UC treatment.

**Supplementary Information:**

The online version contains supplementary material available at 10.1186/s10020-025-01264-9.

## Introduction

Urothelial carcinoma (UC) is the second most common cancer of the urinary system (Lin et al. 2023) and 10 th most common cancer in men worldwide (Saginala et al. 2020). UC exhibits a high incidence rate in Taiwan (Hung et al. 2016) and evolves through two distinct pathways, giving rise to non-muscle-invasive papillary tumors and muscle-invasive tumors (Sanli et al. 2017). The primary treatment options for UC includes transurethral resection (where applicable) followed by chemotherapy (Hannouneh et al. 2023), while emerging treatment includes immune checkpoint blockade medications such as atezolizumab, alone or in combination with chemotherapy (Tran et al. 2021; Chien et al. 2020). Despite these treatments, factors such as the tumor microenvironment, can influence their effectiveness, often resulting in tumor recurrence or metastasis (Sanli et al. 2017; Wolacewicz et al. 2020). In addition, ICB resistance is also observed in UC patients (Snyder et al. 2017), contributing to poor prognosis and adverse side effects. Given these challenges, this is a critical and imperative need to develop alternative treatment strategies that offer better survivability and effective response for UC patients.

A well-coordinated immune system plays a critical role in detecting and eliminating infected, transformed, or tumor cells (Corthay 2014; Gonzalez et al. 2018). Among these defenses, Natural Killer (NK) cells are key agents in combating malignancies and viral infections, primarily through their cytotoxic capabilities (Xia et al. 2021). These cells release granzyme B and perforins to initiate direct cytotoxicity against targets (Wu et al. 2020; Shin et al. 2020; Carlsten and Jaras 2019), which is facilitated by the recognition of MHC class I-related molecules such as MICA, MICB, and NKG2D ligands (NKG2DLs) on the surfaces of tumor cells (Wu et al. 2020; Topham and Hewitt 2009).

NK cells are equipped with a diverse array of activating and inhibitory receptors, which interact with respective ligands to orchestrate a complex network of signals (Nayyar et al. 2019). This network triggers immune responses that can either directly or indirectly induce cytotoxicity, effectively eliminating infected or transformed cells. Crucially, NK cells maintain a delicate balance between the signals from activating and inhibitory receptor-ligand interactions (Wu et al. 2020; Topham and Hewitt 2009; Garcia-Cuesta et al. 2015). When an activating signal overrides inhibitory ones, NK cells are empowered to directly lyse cancer cells (Liu et al. 2022; Bauman et al. 2016).

A crucial activating receptor on NK cells is NKG2D, which plays an essential role in identifying and targeting cancer cells (Garcia-Cuesta et al. 2015). However, cancer cells, including UC, have developed multiple strategies to evade NK cell surveillance (Liu et al. 2022; Quamine et al. 2021; Ferreira-Teixeira et al. 2016; Zhao et al. 2019; Seliger and Koehl 2022; Bauman et al. 2016; Champsaur and Lanier 2010; Siemaszko et al. 2021), including the suppression of NKG2DLs through various mechanisms such as epigenetic silencing, which has been observed in several cancers (Xia et al. 2021; Zhu et al. 2015; Bhat et al. 2019).

Additionally, the effectiveness of NK cells is significantly influenced by their ability to traffic to and infiltrate tumor sites (Yu 2023). Chemokines such as CCL3 play a crucial role in this process by guiding the migration of NK cells toward the tumor environment (Allen et al. 2018). CCL3 enhances the chemotactic response of NK cells, facilitating their accumulation in and penetration of tumor tissues where they can exert their cytotoxic effects more efficiently (Bernardini et al. 2012). In this regard, therapeutics that can restore the expression of NKG2D ligands and chemokines to enhance NK cell infiltration are urgently needed.

Cyproheptadine (CPH), an anti-histamine drug, has been shown to elicit an anti-tumor response in UC (Hsieh et al. 2016). We have previously identified that CPH may act as a novel epigenetic modifier, showing upregulation of several tumor suppressor genes in UC cells (Jou et al. 2021). However, the role of CPH in enhancing NK cell-mediated anti-tumor immune response is currently unknown. In this study, we investigated the role of CPH in this context and explored the underlying mechanisms. Interestingly, we found that CPH, along with an HDAC inhibitor, can restore NK cell-mediated cytotoxicity towards UC cells, likely due to the restoration of NKG2DLs and CCL3.

## Materials and methods

### Cell lines and cell cultures

Urothelial carcinoma cell lines (UMUC3, J82, BFTC905) and parental NK-92 cells were purchased from ATCC (American Type Culture Collection, Manassas VA). UMUC3 and J82 cells were maintained in MEM (Gibco, Waltham, MA) supplemented with 10% fetal bovine serum (FBS), 1µM sodium pyruvate (Gibco), and 1% penicillin/streptomycin (P/S, Invitrogen, Waltham, MA). BFTC905 cells were maintained in RPMI 1640 (Gibco) supplemented with 10% FBS and 1% P/S. The transformed human embryonic kidney cell line (HEK293 T) was maintained in DMEM (Gibco) supplemented with 10% FBS and 1% P/S. MB49 cells were maintained in RPMI-1640 supplemented with 10% FBS, 1% P/S, 1% HEPES (Gibco), 1% L-glutamine (Gibco), 50µM 2-Mercaptoethanol (Merck, Rahway, NJ). All cells were incubated in 37 °C at 5% CO_2_. Briefly, NK-92 was cultured in the Complete RPMI 1640 medium containing RPMI 1640 (ATCC modification, Gibco) supplemented with 15% heat-inactivated FBS, 25 mM HEPES, 1X GlutaMAX, 1% P/S, and 100 U/ml recombinant human IL-2 (PeproTech, Waltham, MA). For routine passage, NK-92 cells were centrifuged at 200 g for 10 min and resuspended in fresh medium at cell density of 2 − 3 × 10^5^ viable cells/mL every 2 to 3 days.

### Cyproheptadine (CPH) and entinostat (ENT) treatment

Cells were cultured in 10 cm plate at a density of 1 × 10^6^ for 48 h. Then, cells were treated with DMSO as control or 12.5µM and 50µM cyproheptadine (CPH, Sigma-Aldrich, St Louis, MO) and 1µM and 2µM Entinostat (MS-275, Selleck Chemicals, Houston, TX) for 48 h. Following CPH and ENT treatment, cells were harvested for RNA-Seq and protein expression analysis.

### Cytotoxicity assay

Cells were treated with different concentrations of CPH or ENT for 48 h. The viability of cells was evaluated by CCK8 assay (Sigma-Aldrich) according to the manufacturer’s instructions. The absorbance at a wavelength of 450 nm was measured using a 96-well EnSpire multimode plate reader (PerkinElmer, Waltham, MA).

### Plasmid construction and transfection

Human ULBP2 coding sequence (741 bp) conjugated with Kozak sequence was amplified by PCR using specific primers (Suppl Table S1) from SV-HUC1. The PCR product was ligated to yT&A vector (Yeastern Biotech, Taiwan) and then sub-cloned into the pIRES2-EGFP (Clontech, San Jose, CA) at Xhol1 and BamH1 restriction enzyme sites. ULBP2 expressing plasmid or empty vectors diluted were transfected into 1 × 10^5^ UMUC3 cells using Lipofectamine 3000 transfection reagent (Invitrogen, Waltham, MA) according to the manufacturer’s protocol. Transfected cells were cultivated with fresh culture medium containing 400 µg/mL Neomycin (G418, Sigma) and replaced every 3 days. Cells were harvested for further experiments after 2-week of selection.

### Knockdown of ULBP2 by ShRNA

The shRNA of ULBP2 was acquired from the RNAi Core Facility (Academia Sinica, Taiwan). Briefly, 293 T cells were transfected with shRNA (TRCN0000056730 or TRCN0000056731), pMDG, and pCMV-dR8.91 using CaCl_2_ transfection method to prepare the shULBP2 lentivirus. Lentivirus-infected BFTC905 UC cells were selected by incubating with 2 µg/mL puromycin (Sigma) for at least 2 days.

### Flow cytometric analysis

Urothelial carcinoma cells or tumor infiltrating lymphocytes (TILs) were incubated in 100 µl of 1X PBS containing and the following antibodies: anti-human ULBP2 (R&D, Minneapolis, MN), APC anti-mouse IgG (BD Pharmingen, Franklin Lakes, NJ), Alexa Fluor^®^ 488 anti-mouse RAE-1ε (R&D), FITC anti-mouse NK1.1 (BD Pharmingen), APC anti-mouse CD45 (Biolegend, SanDiego, CA), PE anti-mouse CD3 (Biolegend), FITC anti-Human CD3 (BD Pharmingen), PE anti-Human CD56 (BD Pharmingen), APC anti-Human NKG2D (CD314, Biolegend, SanDiego, CA) for cell surface markers according to manufacturer’s instructions. After incubation, cells were washed in PBS and analyzed with BD FACS Calibur™ flow cytometer (Becton Dickinson, Franklin Lakes, NJ) or BD Accuri™ C6 Plus (Becton Dickinson).

### Promoter insertion at the *NKG2D* gene by CRISPR knock-in

A synthetic CMV promoter was inserted upstream of the *NKG2D* gene to bypass the endogenous promoter and upregulate the gene expression. We used the CRISPR-mediated promoter knock-in strategy as described in Huang et al. (2020). Briefly, a synthetic DNA template, encoding the CMV promoter flanked by the *NKG2D* homology arms (Suppl Table S2), was purchased as gBlock fragment from IDT-DNA, USA. The DNA template contained silent mutations in the right homology arm (marked by bold sequences) to prevent cleavage by Cas9 that was programmed to target the genomic site. The DNA template was amplified by PCR using primer set (Suppl Table S1) and KAPA HiFi DNA Polymerase kit (Roche, Rahway, NJ). The PCR product was analyzed by DNA gel electrophoresis and validated by Sanger sequencing. The PCR products were purified by QIAquick PCR Purification Kit (Qiagen, Germany) and eluted in nuclease-free H2O (IDT-DNA). The DNA concentration was determined by OD_280nm_ using NanoDrop Lite (Thermo Fisher Scientific, USA) and adjusted to 1 µg/µL in nuclease-free H_2_O and stored at − 20 °C.

CRISPR knock-in was performed by the electroporation of DNA template and pre-assembled Cas9 ribonucleoprotein (Cas9 RNP) into NK-92 cells in a Lonza 4D Nucleofector system as described (Huang et al. 2020). Cas9 RNP and the *NKG2D-*targeting single-guide RNA (guide sequence: TCGGAGGTCTCGACACAGCT) were prepared as described (Lin et al. 2022). After electroporation and recovery, the cells were transferred to a 24-well plate filled with 1 mL of pre-warmed Complete RPMI 1640 medium and maintained by the standard method. To isolate the NKG2D-positive clones, the edited NK-92 cells were single-cell sorted using FACSAria III (BD Biosciences, USA) and seeded in 100 µL of complete RPMI 1640 medium with 100 U/mL of IL-2 in each well of the 96-well round-bottom plate (Techno Plastic Products, Switzerland). Fresh IL-2 was replenished every four days up to 20 days after sorting. The NK-92 single colonies were transferred to a 24-well plate containing 1 mL of the Complete RPMI 1640 medium for further expansion. NKG2D expression was validated by flow cytometry.

### NK cell cytotoxic assay

NK cell cytotoxic assay was performed using Calcein-AM dye as previously described (Huang et al. 2020) In brief, CPH or ENT pretreated cancer cells were resuspended in 1 ml 1xPBS containing 10µM Calcein-AM (BioLegend, San Diego, CA) and incubated at 37⁰C for 30 min. The cells were then washed by culture media for three times and resuspended at the cell density of 1 × 10^5^ cells/ml in RPMI-1640 (ATCC modification). NK-92 cells were pelleted at 200xg for 10 min and resuspended in RPMI-1640 (ATCC modification). One hundred microliters of different cell density of NK-92 cells or primary human NK cells was added to U-bottom 96-well plates (Falcon, corning, NY) for different NK-to-target ratios, followed by addition of one hundred microliter of the stained target cells into each well. The 96-well plate was the centrifuged at 120 g for 3 min to accelerate the contact between NK and target cells. The cells were allowed to co-culture for 4 h at 37◦C incubator. Spontaneous release of Calcein-AM was measured in the absence of NK cells. Maximal release was determined by complete lysis of target cells in RPMI-1640 (ATCC modification) containing 2% Triton-X100. After co-culture, the plates were centrifuged at 120 g for 3 min, and 100 µl of the supernatant was transferred to black 96-well (CSL-30296) plates. The 488/520 values were recorded using FLUOstar Omega (BMG Labtech, Germany) and the cytotoxicity was calculated as follow.

$$\mathrm T\mathrm a\mathrm r\mathrm g\mathrm e\mathrm t\;\mathrm L\mathrm y\mathrm s\mathrm i\mathrm s=\frac{\mathrm E\mathrm x\mathrm p\mathrm e\mathrm r\mathrm i\mathrm m\mathrm e\mathrm n\mathrm t\mathrm a\mathrm l\;\mathrm r\mathrm e\mathrm l\mathrm e\mathrm a\mathrm s\mathrm e-\mathrm s\mathrm p\mathrm o\mathrm n\mathrm t\mathrm a\mathrm n\mathrm e\mathrm o\mathrm u\mathrm s\;\mathrm r\mathrm e\mathrm l\mathrm e\mathrm a\mathrm s\mathrm e}{\mathrm m\mathrm a\mathrm x\mathrm i\mathrm m\mathrm a\mathrm l\;\mathrm r\mathrm e\mathrm l\mathrm e\mathrm a\mathrm s\mathrm e-\mathrm s\mathrm p\mathrm o\mathrm n\mathrm t\mathrm a\mathrm n\mathrm e\mathrm o\mathrm u\mathrm s}\times100$$

### Isolation of primary NK cells

Umbilical cord blood from three normal healthy volunteers was collected and primary natural Killer (NK) cells were isolated from the cord blood mononuclear cells (MNCs) using NK cell Isolation Kit (Miltenyi Biotec, Germany). Primary NK cells were maintained and expanded using SCGM growth media (CellGenix, Germany) supplemented with 100U/ml IL-2 (Pepro Tech, Waltham, MA) and 10U/ml IL-15 (PeproTech, Waltham, MA). The purity of the expanded NK cells were assessed via flow cytometry using Anti-human CD3, and CD56 markers. This study was approved by the Institutional Review Board (IRB) of Taoyuan General Hospital (IRB no.: TYGH109026), Ministry of Health and Welfare, Taiwan. All healthy volunteers received informed consent.

### RNA extraction and expression analysis

RNA was extracted with the TRIzol Reagent (Invitrogen, Waltham, MA) according to manufacturer’s instructions. In brief, after removing cell culture media was removed followed with 1X PBS wash, then the cells were collected in 1 ml TRIzol reagent for RNA extraction. RNA purity were analyzed by spectrophotometry. For expression analysis, 1 µg of the RNA sample was transcribed into cDNA using MMLV Reverse Transcriptase (Episcript, UK) as previously described (Cheng et al. 2019). The mRNA expression in UC cell lines was examined by qPCR using StepOne Real-Time PCR System (Applied Biosystems, Waltham, MA). All RT-PCR primer sequences are shown in supplementary Table S2. The relative gene expression level was determined by comparing the threshold cycle (Ct) of the test gene against the Ct of GAPDH in the given sample.

### Chromatin Immunoprecipitation (ChIP)-qPCR

In brief, 1 × 10^7^ control or experimental cells were cross-linked in 1% formaldehyde solution (Sigma, St. Louis, MO). After neutralizing excess formaldehyde with glycine, fixed cells were collected and sonicated into 500-bp (average) fragments with Diagenode Bioruptor Pico (Diagenode, Denville, NJ). Magnetic Dynabeads (Invitrogen, Waltham, MA), combined with a mixture of anti-human H3K27Ac (Cell Signaling, Danvers, MA), anti-H3 K27me3 (Active Motif, Carlsbad, CA), or rabbit-IgG (Abcam, UK), were used to immunoprecipitate specific crosslinked DNA. Immunoprecipitated DNA-protein complexes cross-links were reversed, and the protein was digested with proteinase K (Invitrogen). DNA fragments were recovered and used as templates for qPCR (Bio-Rad, Hercules, CA) amplification, using specific primers (Supplementary Table S2).

### RNA-seq analysis

RNA was extracted using RNA extraction kit (RNeasy mini kit, Qiagen, Germany) and 1ug of total RNA of each sample was used in duplicates (*n* = 2) for sequencing using Illumina NovaSeq 6000 platform at Genomics LLC, Taiwan. All sequencing data and analyzed for differentially expressed genes and graphs were plotted accordingly. RNA-seq data were deposited into the Gene Expression Omnibus database (accession number: GSE266660).

### In-vivo mouse model

Five-weeks old C57BL/6 mice were purchased from National Laboratory Animal Center, Taiwan. 2 × 10^5^ MB49 UC cells were mixed with matrigel (Corning Matrigel^®^, Corning, NJ) at 2:1 ratio and subcutaneously inoculated to either flank of each mouse. A dose of 5 mg/kg of CPH (Sigma-Aldrich) or 3 mg/kg of ENT (MS-275, Selleck Chemicals) was used as previously described (Hsieh et al. 2016; Smith et al. 2018) and diluted in saline solution and injected intraperitoneally for five days per week for the total period of 2 weeks. 2–5% DMSO was used in the control group. The tumor size was assessed daily by measuring the tumor length (L) and width (W) to determine the volume using the formula V = 0.5 × L × W^2^. At the end of the experiment, the tumor was excised for infiltrated NK cell analysis. Tumor cells were meshed against 0.2µM filter and collected with T-cell medium (TCM) in a centrifuge tube and mononuclear cells were isolated using Ficoll-Paque (Cytiva, Sweden) by centrifuging at 400 g for 20 min at room temp. Then the mononuclear cells were isolated by pooling out the buffy coat layer along with the clear medium and transferred to new centrifuge tube. The cells were centrifuged again at 400 g for 10 min at 4ºC. After centrifugation, supernatant was removed and cells were gently washed using TCM and resuspended in 1 ml of TCM. The resulting cell suspension, containing a count of 5 × 10^5^ cells, were transferred to flow tubes for immune cell staining and FACS analysis. The study protocol involving animal experiments was approved by the Institutional Animal Care and Use committee (IACUC) of the National Chung Cheng University, Taiwan, and performed in accordance with the approved regulations concerning animal experiments.

### Immunohistochemistry

Paraffin-embedded tissue sections were stained with H&E or anti-NK1.1 antibody (Cell Signaling) for NK cells and observed under microscope at 40X resolution and digitally scanned to analyze the results. The scanned regions were observed for NK1.1 stained cells and 4 randomly chosen regions were selected to calculate the amount of infiltrated NK cells by using ImageJ software (Schneider et al. 2012).

### Protein analysis of CCL3 by ELISA

Protein was extracted from MB49 cells treated with CPH or ENT, using 100 µl of tissue extraction reagent (Invitrogen) containing 1X protease inhibitor (Roche) and 1X phosStop (Roche). The amount of CCL3 was measured using Mouse MIP-1 alpha/CCL3 ELISA Kit (Abcam) according to the manufacturer’s protocol.

## Results

### CPH and ENT enhance NK cell-mediated cytotoxicity in UC cells using NK-92 cells

**Fig. 1:**
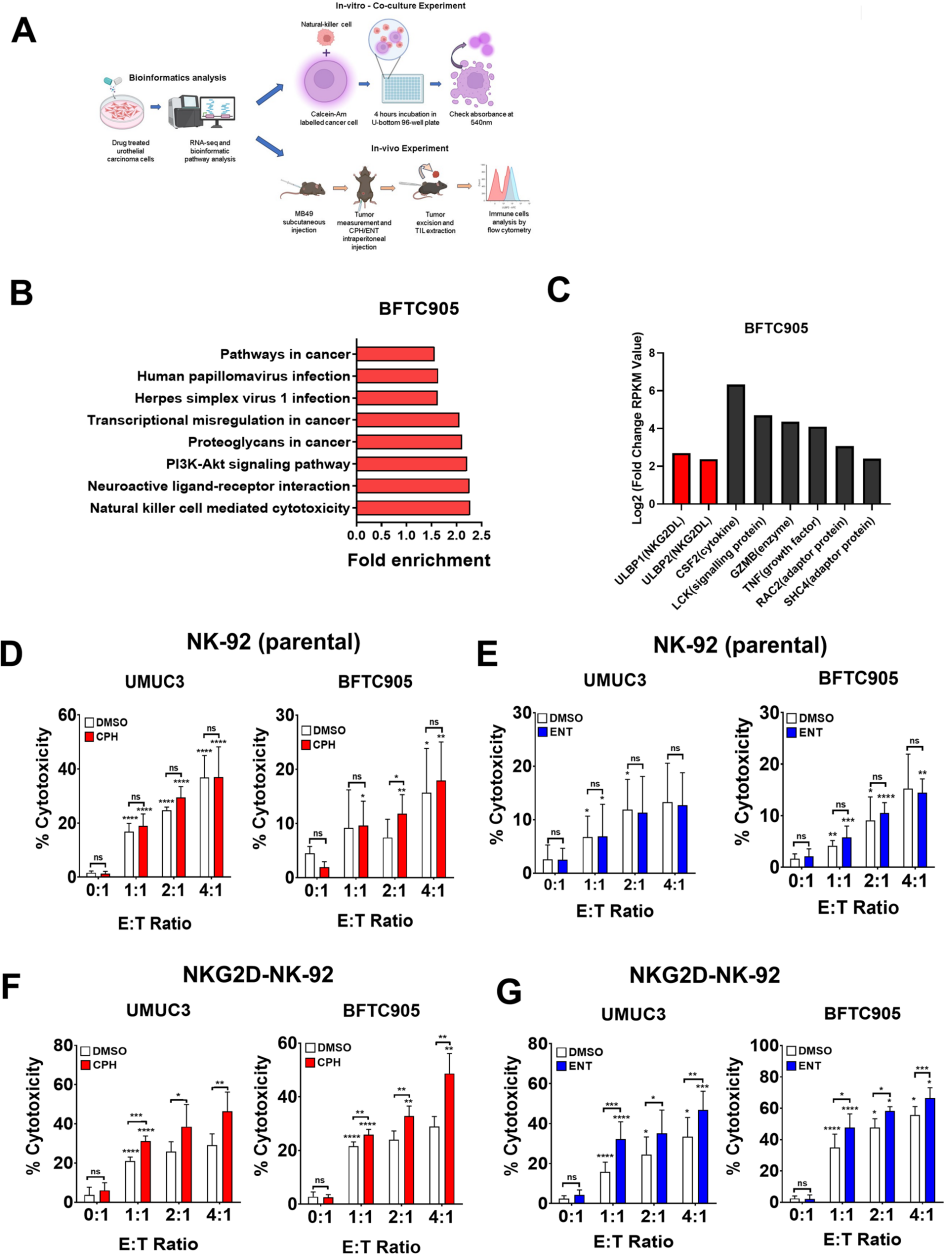
NKG2D NK-92 cells exhibited enhanced NK cell-mediated cytotoxicity in UC cells pretreated with epigenetic drug. **A** schematic representation of the experimental setup. **B** Histogram showing enrichment of upregulated genes associated pathways and **C** list of upregulated genes using RNA-Seq data from BFTC905 UC cells treated with cyproheptadine (CPH). UMUC3 and BFTC905 cells pretreated cyproheptadine (CPH, 50 µM in UMUC3 and 25 µM in BFTC905 cells) or Entinostat (ENT, 2 µM) were co-cultured with either (**D**, **E**) parental NK-92 or (**F**, **G**) NKG2D-NK-92 cells. Bar chart showing cell specific lysis with different effector to target (E: T) ratio as the measure of NK cell-mediated cytotoxicity via calcein-AM assay (For detail, please see material and methods). Interestingly, pretreatment with CPH or ENT only further enhanced the NK cell-mediated cytotoxicity in NKG2D-NK-92 cells (**F**, **G**), but not parental NK-92 cells (**D**, **E**). Each error bar represents mean ± SD from triplicates. The significance is calculated by unpaired T-test. ****P < 0.0001; *** *P* < 0.0005; **P < 0.01; *P < 0.05. Individual asterisk shown on each bar compares to the previous E to T ratio bar of each group

NK cells are pivotal to our immunity due to their potent cytotoxicity against cancer cells (Shin et al. 2020). As we previously demonstrated the anti-tumor activity of CPH in UC cells (Hsieh et al. 2016), we then explored the potential mechanism of CPH in this context. Interestingly, pathway analysis, using our previous RNA-Seq dataset from BFTC905 UC cells treated with CPH (Jou et al. 2021), identified enrichment of genes related to NK cell-mediated cytotoxicity (Fig. 1A, B). Specifically, ULBPs were observed to show the highest upregulation amongst other genes in this pathway after CPH treatment (red bar, Fig. 1C, Suppl Table S3). Motivated by these results, we investigated whether CPH could enhance NK cell-mediated cytotoxicity against UC cells. In addition, we compared the effect of CPH to entinostat (ENT), a known HDAC inhibitor, as our previous study suggested that CPH may possess HDAC inhibitory activity.

UMUC3 and BFTC905 cells were first pretreated with either CPH or ENT, at concentrations that maintained at least 70% viability, as demonstrated by the CCK8 assay (Suppl Fig S1A, B). Indeed, pretreatment of the drugs did not significantly affect the viability of the UC cells (Fig. 1D, E, E: T ratio 0:1). The drug-treated cells then co-cultured with NK-92 NK cells (Huang et al. 2020), which was confirmed to have potent cytotoxic ability against MHC-I deficient K562 cells (Suppl Fig S2A) (Song et al. 2020). Although increasing effector to target (E: T) ratio demonstrated an increased cell-specific lysis in UMUC3 and BFTC905 cells, pre-treatment with CPH (Fig. 1D) and ENT (Fig. 1E) did not significantly enhance the cell-specific lysis in these cells.

These results might be due to low expression of the NKG2D receptor in NK-92 cells, potentially limiting their ability to bind with ULBPs-expressing tumor cells. Indeed, flow cytometry confirmed that NKG2D was not highly expressed in NK-92 cells (Suppl Fig S3). To address this issue, we enhanced the NKG2D expression in NK-92 cells (referred to as NKG2D-NK-92) by CRISPR genome editing to knock-in an CMV promoter upstream of NKG2D. The synthetic promoter bypassed the endogenous promoter and upregulated the NKG2D expression (Suppl Fig S3). Co-culture experiments with NKG2D-NK-92 cells demonstrated a significant enhancement in NK cell-mediated cytotoxicity against K562 (Suppl Fig S2B), UMUC3, and BFTC905 cells treated with CPH (Fig. 1F) or ENT (Fig. 1G). Fluorescence microscopy images further supported these findings, showing a decrease in target cell density indicative of NK cell-mediated cytotoxicity (Suppl Fig S4).

### CPH and ENT enhance NK cell-mediated cytotoxicity in UC cells using primary NK cells

**Fig. 2:**
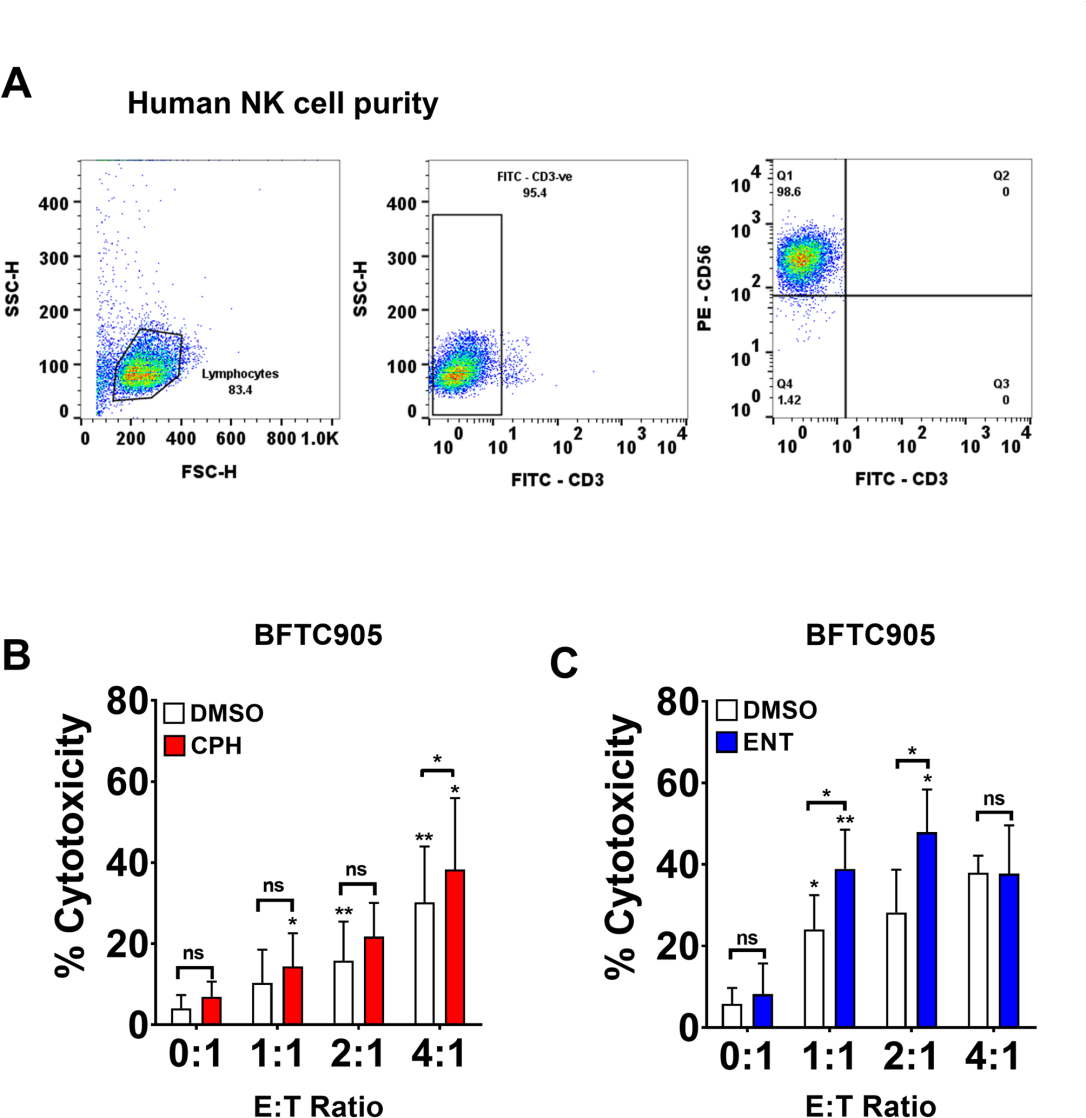
Primary human NK cells exhibited enhanced NK cell-mediated cytotoxicity in UC pretreated with epigenetic drug. Primary NK cells were isolated from cord blood of three health donor. **A** Representative flow cytometry analysis showing purity of expanded primary human NK cells using CD3^−ve^CD56^+ve^. Left panel: lymphocyte population; middle panel: CD3^−ve^ population; right panel: CD3^−ve^CD56^+ve^ population. Bar chart showing cell specific lysis with different E:T ratio as the measure of NK cell-mediated cytotoxicity using expanded primary NK cells, via calcein-AM assay, in BFTC905 cells pretreated with (**B**) 25 µM CPH or (C) 2 µM ENT. Representative experiments of human primary NK cells isolated from the cord blood PBMCs of one healthy donor’s were shown. Each error bar represents mean ± SD from triplicates. The significance is calculated by unpaired T-test. ****P < 0.0001; *** *P* < 0.0005; **P < 0.01; *P < 0.05. Individual asterisk shown on each bar compares to the previous E to T ratio bar of each group

Next, we investigated whether similar cytotoxic function can be observed using human primary NK cells. Considering this, we isolated and expanded human primary NK cells from peripheral blood mononuclear cells (PBMCs) derived from umbilical cord blood (Suppl Fig S5). Flow cytometry analysis of expanded primary human NK cells demonstrates a high purity, with 96–98% of the cells, being identified as CD3^−ve^ CD56^+ve^ NK cells (Fig. 2A). 

Co-culture experiment demonstrated that primary human NK cells exhibit enhanced cytotoxicity against UC BFTC905 cells, treated with CPH (Fig. 2B) and ENT (Fig. 2C). Taken together, these results suggest that epigenetic treatment in UC cells can enhance the NK cell-mediated anti-tumor immune response in both NKG2D-NK-92 cells and primary NK cells.

### CPH and ENT restore the expression of ULBP2 in UC cells

**Fig. 3:**
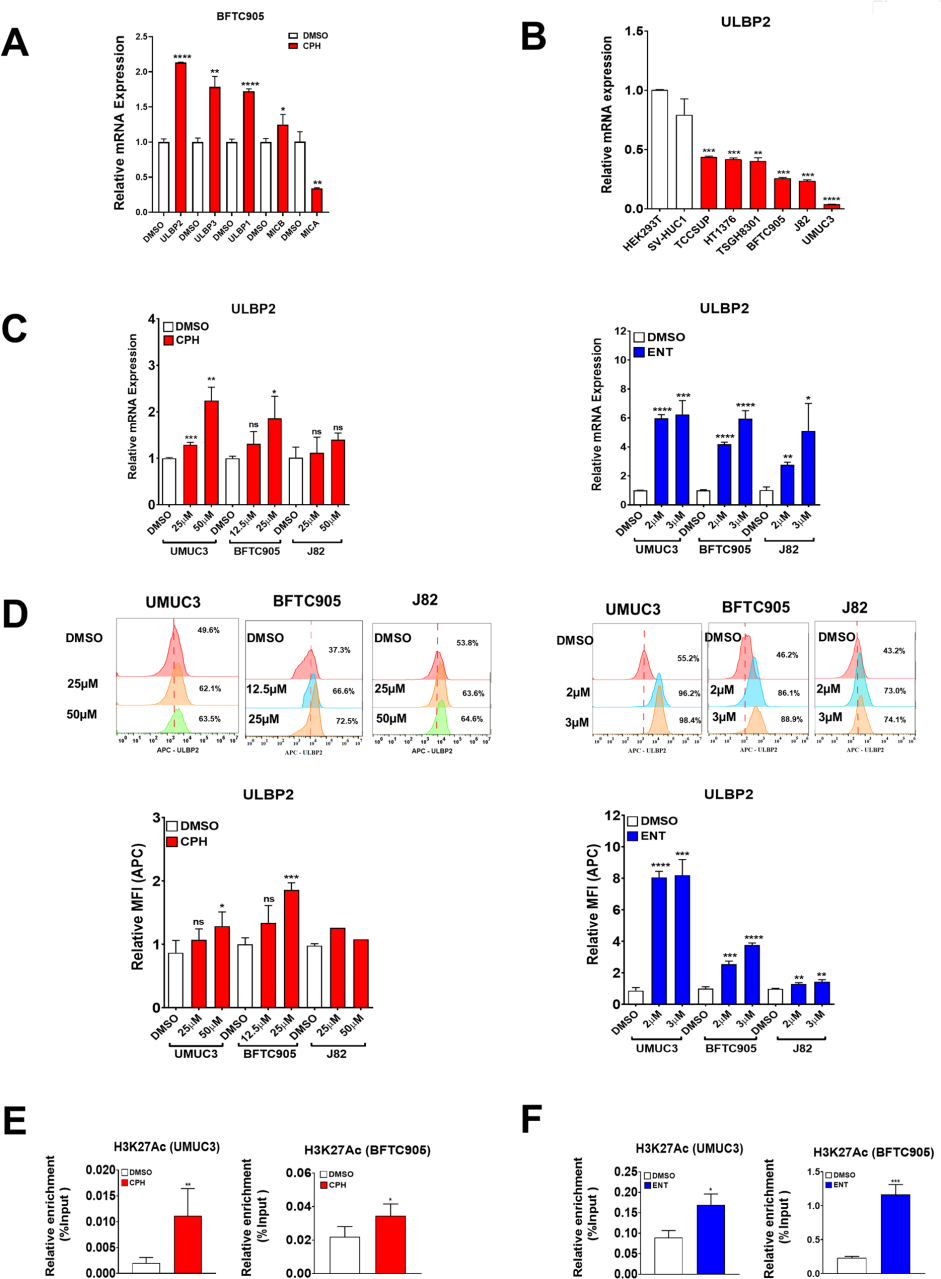
Restoration of NKG2D ligands in urothelial carcinoma treated with CPH or ENT. **A** Bar chart showing the relative mRNA expression of NKG2DLs in BFTC905 cells treated with CPH 25µM for 48 h using qRT-PCR. **B** Relative expression of ULBP2 in HEK293T, SV-HUC1 and a panel of UC cell lines by quantitative RT-PCR. Expression of ULBP2 in UC cells treated with CPH (left) or ENT (right) by (**C**) quantitative RT-PCR and (**D**) flow cytometry analysis. Enrichment of H3K27 Ac at the promoter of ULBP2 was determined in UC cells treated with (**E**) CPH (25 µM in BFTC905, 50µM in UMUC3) or (**F**) ENT (2µM) as determined by ChIP-qPCR. Each error bar represents mean ± SD from triplicates. The significance is calculated by unpaired T-test. ****P < 0.0001; *** *P* < 0.0005; **P < 0.01; *P < 0.05

In light of the enriched NK cell-mediated cytotoxicity pathway in BFTC905 cells treated with CPH (Fig. 1B, C), we then investigated the expression of genes involved in NK cell-mediated cytotoxicity in BFTC905 UC cells. Specifically, both RNA-Seq and qRT-PCR showed that treatment with CPH indeed upregulate the expression of the NKG2DLs, except for MICA, with the highest expression level of ULBP2, compared to other NKG2DLs in BFTC905 cells (Fig. 3A, Suppl Table S3, Suppl Fig S6). Previous study demonstrated that ULBP2 has been reported to be epigenetically suppressed in various cancers (Lopez-Soto et al. 2009). In this regard, we first examined mRNA levels of ULBP2 in a panel of UC cells in comparison with SV-HUC (immortalized normal bladder epithelial cells) and HEK-293T (immortalized normal kidney cells). Our results indicated a downregulation of ULBP2 in UC cells, as compared to SV-HUC1 and HEK293T cells (Fig. 3B). We then examined whether ULBP2 can be epigenetically restored in UC. Our results showed that treatment with CPH or ENT can upregulate ULBP2, in both mRNA (Fig. 3C) and protein (Fig. 3D), in UMUC3, BFTC905, and J82 UC cells.

Further experiments demonstrated that ULBP2 is epigenetically silenced by histone modification, but not DNA methylation (Suppl Fig S7), as CPH (Fig. 3E) and ENT (Fig. 3F) restored the enrichment of the active histone marks H3K27Ac in UMUC3 and BFTC905 cells as demonstrated by ChIP-qPCR. Additionally, a decrease enrichment of the repressive histone mark H3K27me3 on the ULBP2 promoter was also observed in in CPH and ENT treated cells (Suppl Fig S8). Taken together, these results suggested that ULBP2 is epigenetically silenced by histone modifications and can be restored by epigenetic reprogramming following CPH and ENT treatment, further confirming that CPH is an epigenetic modifier.

### Overexpression of ULBP2 enhanced NK cell-mediated cytotoxicity in UC cells

**Fig. 4:**
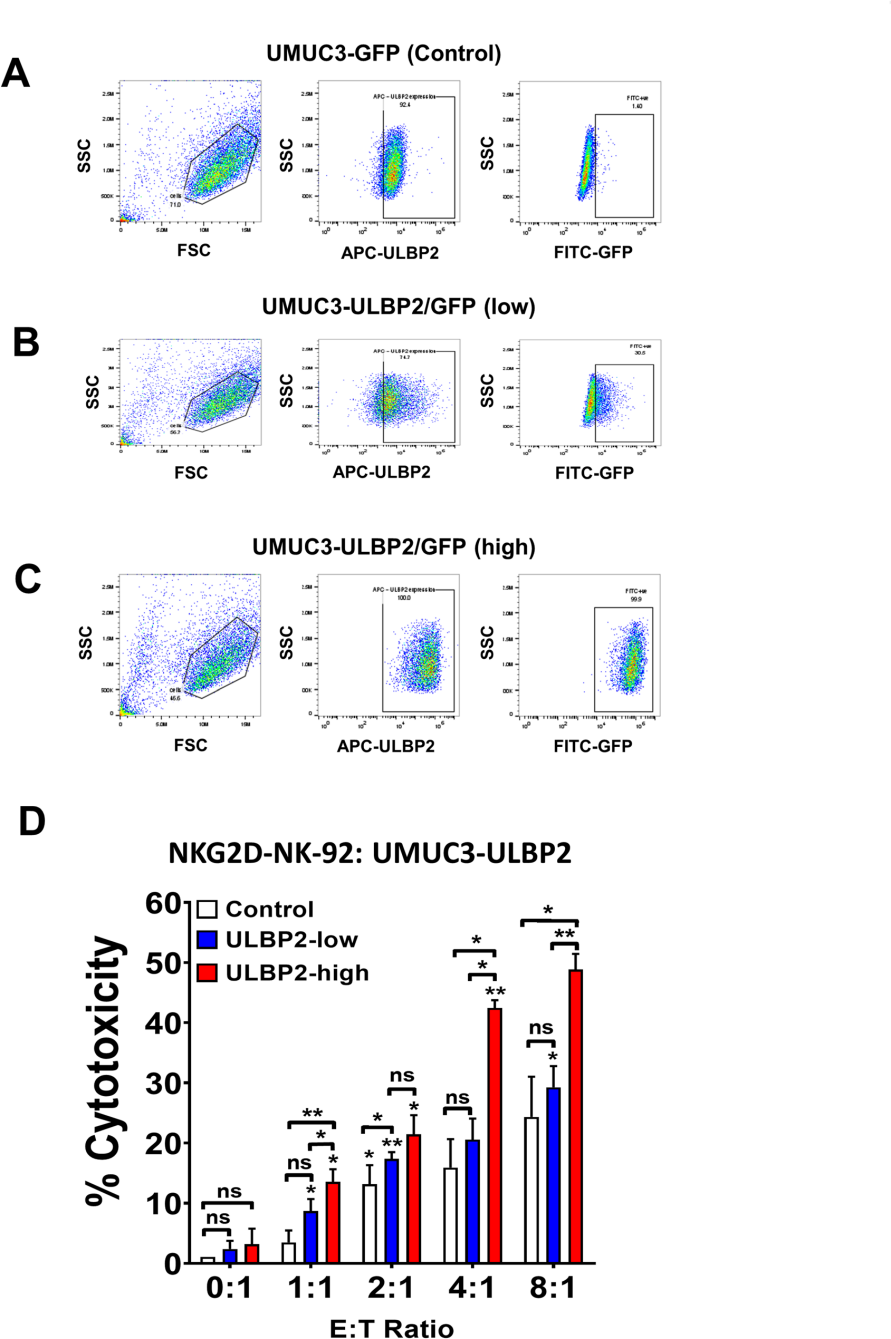
Enhanced NK cell-mediated cytotoxicity in ULBP2 overexpressing UC cells. Flow cytometry analysis of UMUC3 cells expressing (**A**) control, (**B**) low, or (**C**) high level of ULBP2, as determined by ULBP2 (middle panel) and GFP intensity (right panel). **D** Bar chart showing cell specific lysis via calcein-AM assay with different E:T ratio of NKG2D-NK-92 cells vs. UMUC3 with different expression level of ULBP2 (white: control, no expressing; blue, low expression; red: high expression) in BFTC905 cells. Each error bar represents mean ± SD from triplicates. The significance is calculated by unpaired T-test. ****P < 0.0001; *** *P* < 0.0005; **P < 0.01; *P < 0.05. Individual asterisk shown on each bar compares to the previous E to T ratio bar of each group

The above experiments demonstrated an augmentation in NK cell-mediated cytotoxicity following epigenetic treatment in UC cells. To confirm whether the interaction between ULBP2 and NKG2D predominantly underlies NK cell-mediated cytotoxicity in UC cells, we overexpressed ULBP2 in UMUC3 cells and separated them into two groups based on the ULBP2 and GFP protein expression levels (Fig. 4A-C). RT-PCR experiment also confirmed that these cells expressed high level of ULBP2 as compared to CPH-treated UC cells (Suppl Fig S9). As expected, when co-culturing with NKG2D-NK-92 cells, UMUC3 cells with higher expression of ULBP2 (Fig. 4D, red) showed higher cell-specific lysis, compared to cells with lower expression of ULBP2 (Fig. 4D, blue) or GFP control (Fig. 4D, white). These results confirmed that ULBP2-NKG2D interaction triggers NK cells to kill tumor cells directly. 

To further support this notion, we generated ULBP2 knock-down (KD) BFTC905 cells using shRNA. Quantitative RT-PCR (Suppl Fig S10 A, B) and flow cytometry (Suppl Fig S10 C, D) confirmed the down-regulation of ULBP2 in the KD cells, even after treatment with CPH or ENT. Unexpectedly, upon co-culture with NKG2D-NK-92 cells, ULBP2 KD BFTC905 cells did not show a significant effect on the lysis after treatment with CPH (Suppl Fig S10E) and ENT (Suppl Fig S10 F), as compared to scramble control. These results suggested that epigenetic treatment may upregulate targets other than ULBP2 in eliciting NK cell-mediated cytotoxicity in UC cells.

### CPH and ENT inhibit tumor progression in syngeneic mice model

**Fig. 5:**
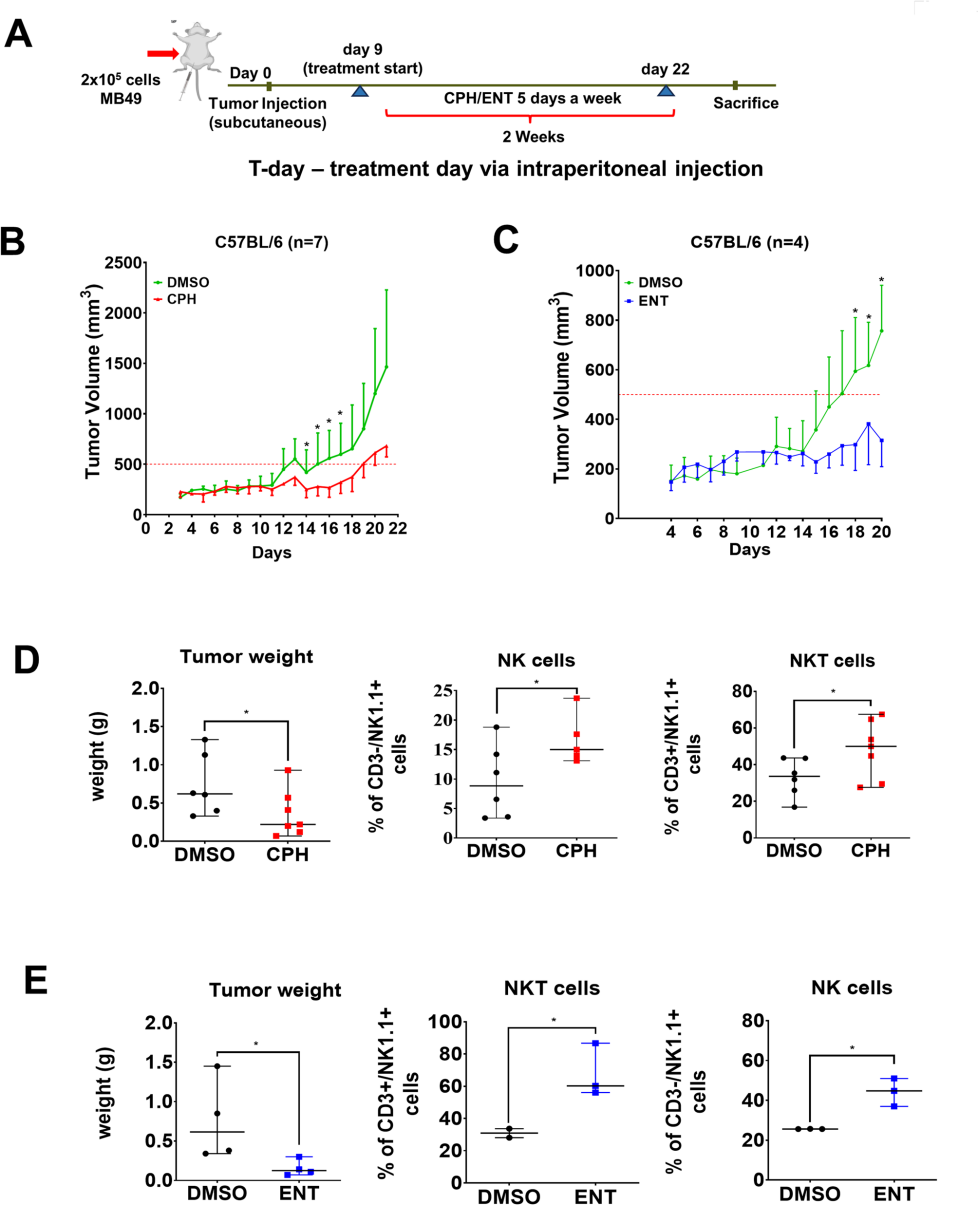
Epigenetic treatment delays tumor progression in syngeneic tumor mice model. **A** Schematic representation of the treatment plan. In brief, MB49 UC cells were injected subcutaneously into C57BL/6 mice. Eight days after the tumor inoculation, 5 mg/kg of CPH or 3 mg/kg of ENT were injected i.p. 5 days per week for 2 weeks. Tumor volume was measured daily. Tumor volume of mice treated with (**B**) CPH (*n* = 7/group) or (**C**) ENT (*n* = 4/group) was shown. Dot plot showing tumor weight, infiltrated CD3-ve NK and CD3 + ve NKT cells, as determined by flow cytometry, in mice treated with (**D**) CPH or (**E**) ENT, respectively. The significance is calculated by unpaired T-test. *P < 0.05

**Fig. 6:**
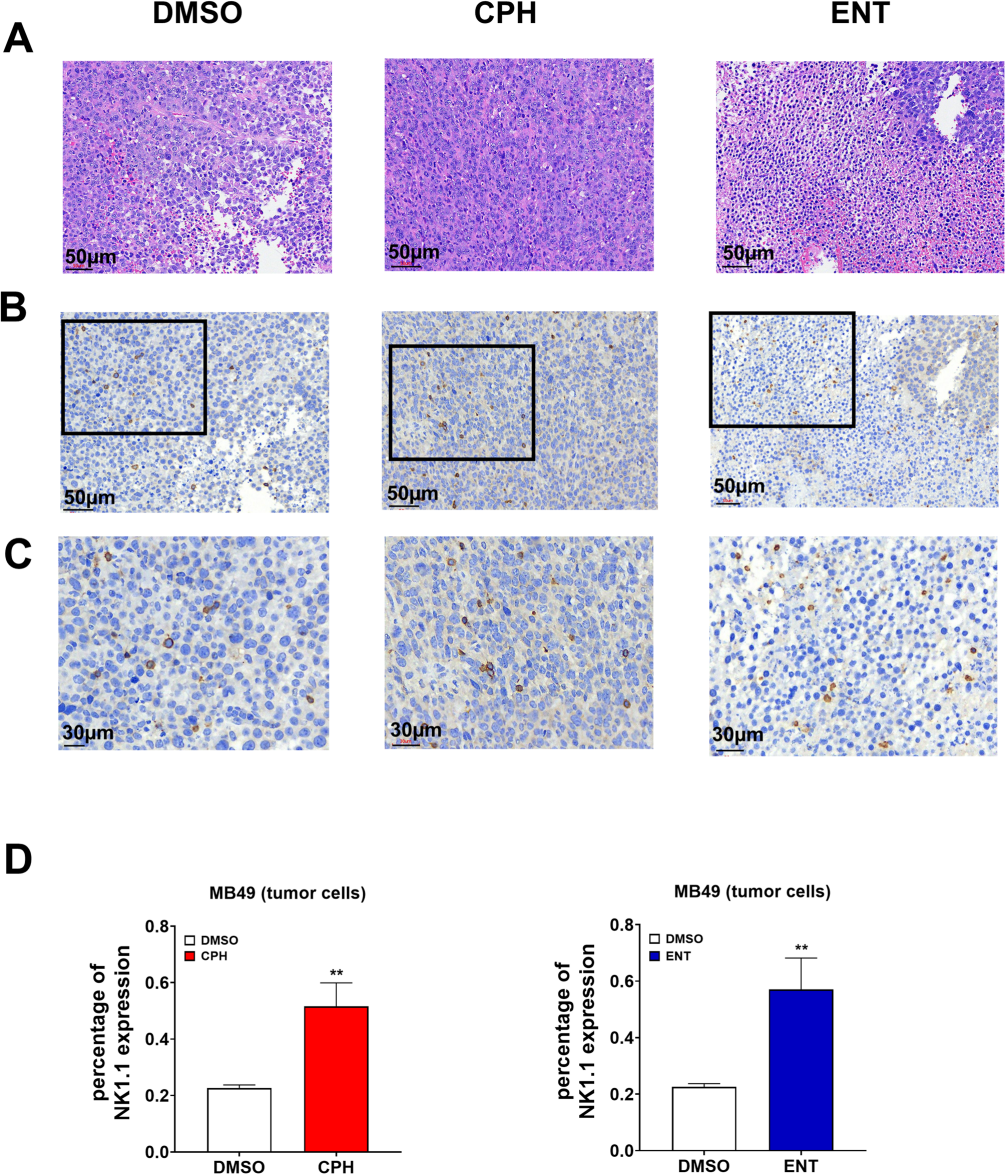
Infiltration of NK cells in tumor microenvironment of syngeneic tumor mice model. **A** Representational illustration of HE stained sample of tumor tissues. **B** IHC stained of NK1.1 showing infiltrated NK cells in tumor microenvironment in dark brown colored cells. The boxed region is enlarged and shown in (**C**). **D** Bar graph showing percentage of infiltrated NK cells in tumor microenvironment. Infiltrated cells were determined using imageJ software. The significance was calculated by unpaired T-test. **P < 0.01

To examine the effect of CPH and ENT in eliciting NK cell-mediated cytotoxicity in UC cells in vivo, we performed a syngeneic mice tumor model using murine MB49 UC cells (Figs. 1A and 5A). Treatment with CPH (Fig. 5B) or ENT (Fig. 5C) inhibited the growth of MB49 tumors in wild-type B6 mice. The reduced tumor growth is probably due to the infiltration of NK, NKT cells in the tumor microenvironment as analyzed by FACS (Fig. 5D, E, Suppl Fig S11 A) and immunohistochemistry (Fig. 6-C). Unfortunately, we observed no significant increase in infiltrated CD8 + T cells in CPH or ENT treatment group (data not shown).

**Fig. 7:**
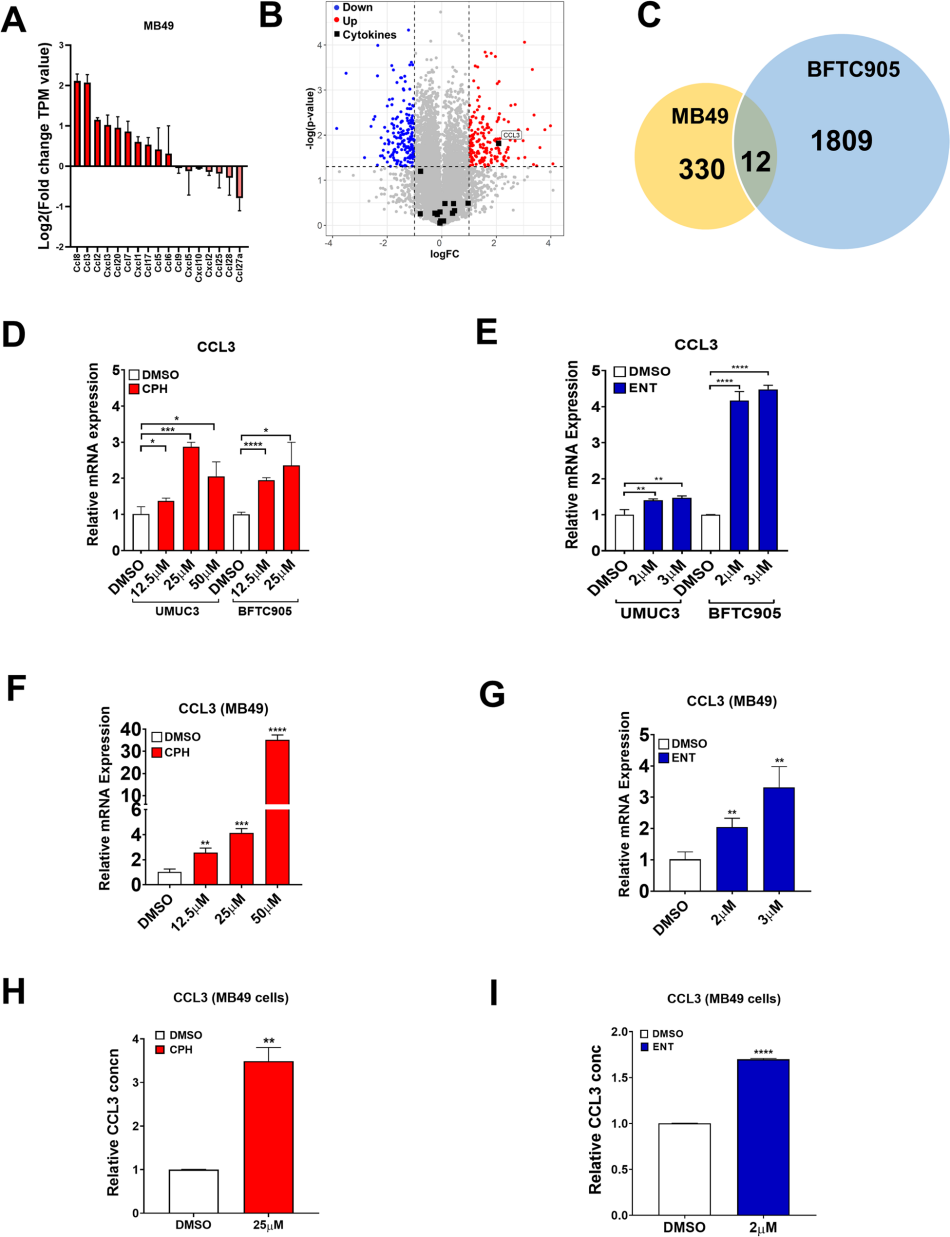
CPH treatment increases the expression of CCL3 in UC cells. **A** Histogram showing expression of chemokines in MB49 cells treated with CPH as determined by RNA-Seq. **B** Volcano plot representing differentially expressed genes of MB49 cells treated with CPH. **C** Venn diagram representing differential upregulated genes and 12 common genes in MB49 cells and BFTC905 cells treated with CPH. Relative expression level of CCL3 mRNA in (**D**, **E**) human and (**F**, **G**) mice UC cells treated with (**D**, **F**) CPH or (**E**, **G**) ENT, as determined by quantitative RT-PCR. Relative protein expression of CCL3 in MB49 parental cells treated with (**H**) CPH and (**I**) ENT is quantified by ELISA. Each error bar represents mean ± SD from triplicates. The significance is calculated by unpaired T-test. ****P < 0.0001; *** *P* < 0.0005; **P < 0.01; *P < 0.05

Moreover, we investigated the differentially expressed genes in CPH-treated MB49 cells utilizing RNA-seq. Unexpectedly, we did not observe any upregulation of NKG2DLs in CPH-treated MB49 cells (Suppl Fig S11B). Remarkably, the analysis revealed an elevation in the expression of chemokines among the CPH-induced upregulated genes with a particularly notable increase observed in the CCL3 expression (Fig. 7A, B), which has been shown to recruit NK cells and augment the production of IFNγ (Allen et al. 2018). 

Intriguingly, upon scrutinizing the commonly upregulated genes among CPH-treated MB49 (330 genes) and BFTC905 cells (1809 genes), we identified CCL3 as the only chemokine among the 12 common upregulated genes (Fig. 7C, Suppl Table S4) following CPH treatment across these two species. To confirm these results in human and mouse UC cells, treatment with CPH (Fig. 7D, F) and ENT (Fig. 7E, G) can indeed upregulate CCL3 in BFTC905, UMUC3 and MB49 cells. In addition, treatment of CPH (Fig. 7H) and ENT (Fig. 7I) induced an upregulation of CCL3 protein level as determined by ELISA assay. Taken together, these results suggest that CPH and ENT may exert their influence on additional pathways to recruit NK cell in the tumor microenvironment to elicit NK cell-mediated cytotoxicity in UC. 

## Discussion

In a variety of carcinomas, we and the others have demonstrated that CPH exhibits significant anti-tumor activity through multiple pathways (Jou et al. 2021; Feng et al. 2012; Hsieh et al. 2017). Recent studies have also unveiled an epigenetic mechanism underlying its anti-neoplastic action (Jou et al. 2021; Takemoto et al. 2016). Furthermore, previous study has indicated that CPH could be a novel agent that enhances the efficacy of HDAC inhibitors in eradicating cancer cells (Paoluzzi et al. 2009), suggesting that CPH may be an epigenetic modifier to exert its anti-tumor effects. In this context, our current study explored the potential mechanism behind the anti-tumor effect of CPH in UC. Our findings, both in vitro and in vivo, indicate that CPH, as well as entinostat (ENT), can enhance the anti-tumor immune response mediated by NK cells.

NK cells play a crucial role in anti-tumor innate immune response (Bernardini et al. 2012) and exert their function by a balance between inhibitory and activating receptors, with NKG2D being one of the most potent activating receptor expressed by NK cells (Ke et al. 2016). However, cancer cells develop several mechanisms, such as downregulation of NKG2D ligands (NKG2DL), to evade the NK cell-mediated immunity (Bauman et al. 2016; Zhou et al. 2020; Peng et al. 2022). In this regard, previous studies have emphasized the significance of restoring NKG2DLs to enhance NK cell-mediated cytotoxicity in various cancer types, including breast cancer (Shen et al. 2017) and epithelial tumor cells (Lopez-Soto et al. 2009). For example, ULBP1, a NKG2D ligand (NKG2DL), has been found to be downregulated in esophageal cancer (Tang et al. 2024). Additionally, other well-characterized NKG2DLs, such as MICA/B was also found to be epigenetically silenced in liver (Bugide et al. 2018), and ovarian cancer (Lopez-Soto et al. 2009). Notably, several studies have demonstrated that epigenetic drug treatments can strengthen NK cell-mediated anti-tumor immune response, particularly due to the restoration of NKG2DLs in human cancer (Bugide et al. 2018; Lu et al. 2022). Specifically, treatment with HDAC inhibitor has been shown to enhance NK cell cytotoxicity in solid tumor, by upregulating MICA/B expression (Armeanu et al. 2005).

Consistent with these findings, our study demonstrated that treatment with CPH or ENT, an HDAC inhibitor, elicited NK cell-mediated immune response in UC cells, both in vitro and in vivo. This effect was achieved through the restoration of ULBP2, a NKG2DL, and the NK-trafficking chemokine CCL3. We also showed that upregulation of ULBP2 by CPH in human UC cells is dependent on epigenetic modifications. This supports previous study indicating that NKG2DLs, including ULBP2, are epigenetically silenced by HDAC3, facilitates NK immune evasion (Lopez-Soto et al. 2009). Notably, CPH and ENT treatment not only restores the enrichment of H3K27Ac but also suppresses the repressive H3K27me3. This effect is likely mediated indirectly through the disruption of HDAC-EZH2 interactions or altered chromatin accessibility following HDACi treatment, which interferes with the recruitment or stabilization of EZH2 at gene promoters and promotes transcriptional activation (Adamik et al. 2017; Kumar et al. 2014).

Interestingly, our observation revealed that treatment with CPH upregulated several chemokines including CCL3. Previous studies have emphasized the critical role of CCL3 in facilitating NK cell migration to tumor sites (Bernardini et al. 2012). For example, in an animal model of bladder cancer, treatment with EZH2 inhibitor enhanced the infiltration of NK cells into the tumor microenvironment, through upregulation of CCL3. Thus, suggests that epigenetic treatment can restore the expression of NK-trafficking chemokine, leading to the infiltration of NK cells to the tumor microenvironment. However, further experiments are required to confirm the role of CPH-induced epigenetic modifications in the expression of CCL3.

NK cells play a crucial role in cancer immunotherapy due to their innate ability to identify and eliminate malignant cells. However, the limited availability and variability of primary NK cells pose significant challenges for consistent research and therapeutic applications. To address this issue, we developed a new protocol with lower amounts of IL-2 and IL-15, as compared to other studies (Nakazawa et al. 2023; Cho and Campana 2009), to expand the primary NK cells. Nevertheless, in this study, we utilized NK-92 cell line, which is derived from a patient with non-Hodgkin’s lymphoma (Gong et al. 1994), which is widely used in experimental research (Klingemann 2023) or in clinical applications (Arai et al. 2008). Notably, NK-92 cells can be efficiently expanded and demonstrate strong antitumor efficacy (Reindl et al. 2020). However, the expression of surface molecules in NK-92 may be affected by culturing conditions that differ from the original protocol using horse serum (Klingemann 2023). We, therefore, suspect that the low NKG2D expression observed in our NK-92 cells is due to prolonged culture in RPMI supplemented with FBS. These observations highlight the need to engineer NK-92 cells to express enhanced or synthetic receptors capable of robust, direct cytolysis of target cells. Research groups, including Ke et al. (2016) and Wang et al. (2017), have demonstrated efforts to overexpress NKG2D in NK-92 cells to enhance their functionality. Such approaches hold promise in overcoming the limitations associated with prolonged culture and compromised NK cell functionality.

Immunotherapy targeting PD-1 or PD-L1 has demonstrated improved overall survival in human cancers. For example, a phase III clinical trial demonstrated that UC patients treated with Avelumab showed improved overall survival (Powles et al. 2020). However, this treatment is effective in only a subset of patients, likely due to insufficient immunocyte infiltration, characteristic of “cold tumors” (Lin et al. 2021). Numerous studies have highlighted the crucial role of restoring CD8 + T-cell infiltration, or converting tumors from “cold” to “hot”, to enhance the efficacy of such immunotherapies (Kumar et al. 2021; Miller, 2019 #108). Nevertheless, the significance of NK cells in this context has been underestimated, as highlighted by a recent study where Hsu et al. (2018) demonstrated that immune checkpoint therapy elicits a strong NK cell response essential for the therapeutic effects of immunotherapy (Hsu et al. 2018). Our results suggest that treatment with CPH may bolster the effectiveness of immunotherapy in human cancer by enhancing NK cell infiltration.

Additionally, a recent study reported that allergy, through histamine action, can promote tumor growth and resistance to immune checkpoint blockade in both mouse models and human cancers. Treatment with fexofenadine, an antihistamine, was found to restore tumor sensitivity to immunotherapy (Li et al. 2022). This suggests that antihistamines, including CPH, may play a role in enhancing the effects of immunotherapy, warranting further investigation.

There are several limitations in this study. First, we did not perform depletion experiments to confirm the contribution of NK cells and CCL3 in the anti-tumor activity in vivo. Second, the potential anti-tumor effects of CPH or ENT as standalone treatments at the current dosage have not been thoroughly investigated in vivo. Future studies involving immunocompromised mice are necessary to further evaluate the anti-tumor efficacy of CPH and ENT in vivo. Alternatively, pharmacokinetics (PK) and pharmacodynamics (PD) studies could be conducted to determine the effective in vivo dosage of these compounds, thereby allowing for a more accurate estimation of the corresponding effective dose in vitro.

**Fig. 8:**
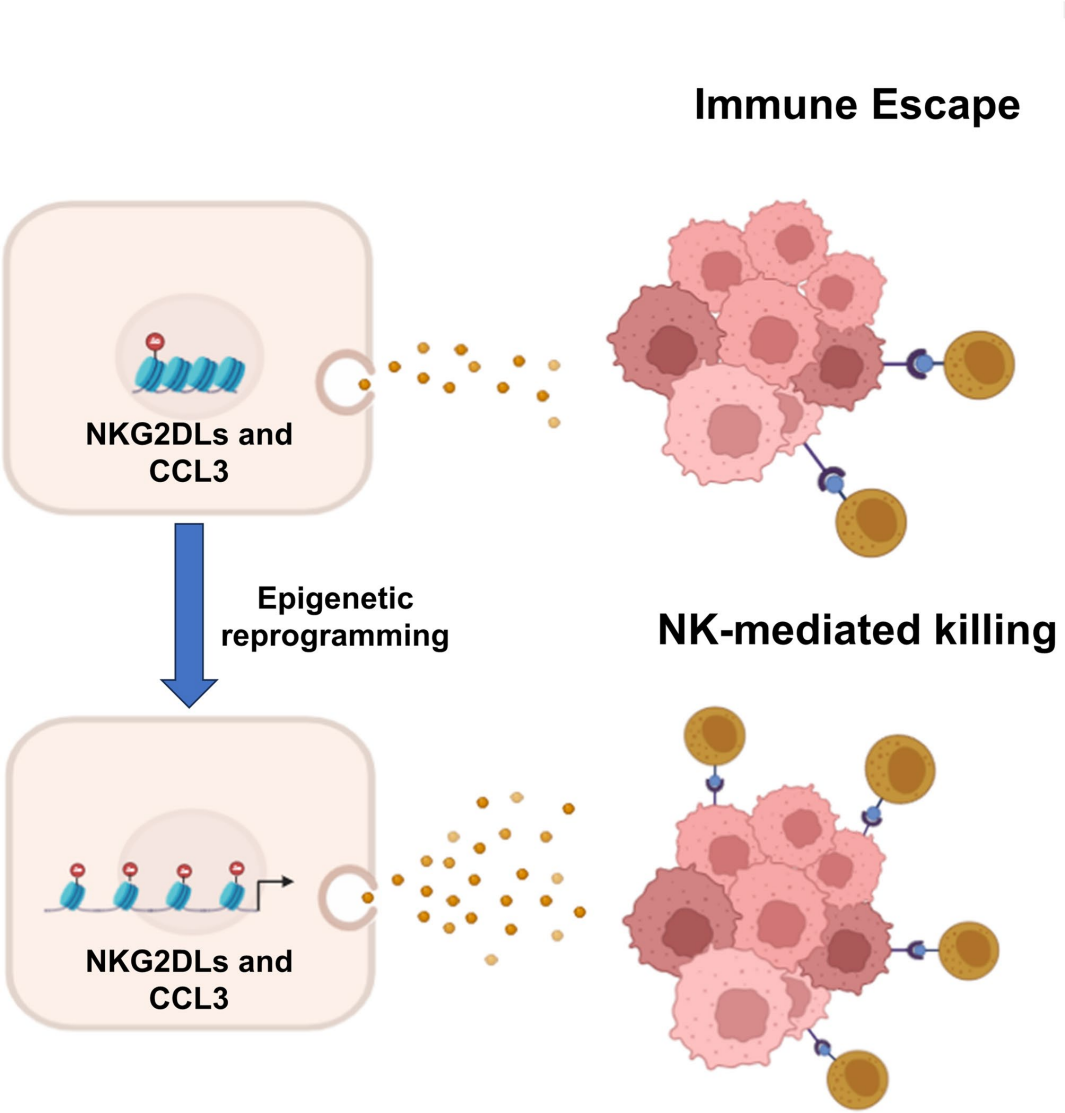
The schematic model depicts that CPH or ENT treatment reprograms the epigenome resulting in the upregulation of NKG2D ligands and CCL3 to enhance the NK cell-mediated cytotoxicity in UC

In summary, our studies have demonstrated that both CPH and ENT exhibit anti-tumor activity by enhancing NK cell-mediated cellular cytotoxicity in UC cell lines and syngeneic tumor mouse models. This effect is attributed to the epigenetic restoration of NKG2DLs and the upregulation of CCL3, leading to increased NK cell infiltration into the tumor microenvironment (Fig. 8). Targeting the epigenetic machinery with agents such as CPH or HDAC inhibitors may offer novel therapeutic strategies to enhance the effectiveness of immunotherapy in human cancers. Further study is warranted to explore the full potential of CPH as an epigenetic modifier in cancer immunotherapy.

## Supplementary Information

Supplementary Material 1.

Supplementary Material 2.

Supplementary Material 3.

## Data Availability

The RNA-Seq data have been deposited in the Gene Expression Omnibus (GEO) database, under accession number GSE266660.
