# Supplementary material for: Epigenetic modulation elicits an NK cell-mediated immune response in urothelial carcinoma
Source: Mol Med. 2025 Jun 24;31:247. doi: 10.1186/s10020-025-01264-9 (PMC12186328; doi:10.1186/s10020-025-01264-9)
Supplement: Supplementary file 1 — Supplementary Material 1. [file 10020_2025_1264_MOESM1_ESM.pdf]

Table S1 Synthetic DNA template, encoding the CMV promoter flanked by the *NKG2D* homology arms

NKG2D-targeting sgRNA guide sequence: TCGGAGGTCTCGACACAGCT

CMV DNA template (Yellow: homology arms; blue: CMV promoter; green: silent mutations):

ATTAAAATGCACTAGAAAAGGACATTCAACAAAAGTGCATATAACTTACCTGAGTTTAGCTTGCAGAAAG
AAAACTAAATTATTGCCAGATAAAATAAACAACTGGAAACACTTGATCTTAGCTAAAAGGCCAAGAAGCA
ATAAACGACTATAAACAGAAAACTGGGTGTGTTGTTATCTAATATCAGTAAAGTAAAATATTGGTATTAA
ATATCTCAATGTAGGTGAATTAATAGGTTCATTATATTGTTATGACATTTTGAAGTAATTGGCGATTATT
TTACAACCAAGAGAAATGTATAAAATTAATTTTGCCTGGCTTTATGTGAATTGTCATTTCTATTCTTTTC
TTAAAGGCATGCACAGGGGAAAAGTTTCTGCATTTTACTTTTCTATTCAAAATATTTTATCTCACCACGG
GAAAGAGAACAAAAAACAAATGCCCAGTTTTATTTTATTCAGGAATCCTTTGTGCATTGAAGACTTTAGA
TTCCTCTCTGCGGTAGACGTGCACTTATAAGTATTTGCCGCATGCCGTATACTCCGGAATATTAATAGGA
CATTGATTATTGACTAGTTATTAATAGTAATCAATTACGGGGTCATTAGTTCATAGCCCATATATGGAGT
TCCGCGTTACATAACTTACGGTAAATGGCCCGCCTGGCTGACCGCCCAACGACCCCCGCCCATTGACGTC
AATAATGACGTATGTTCCCATAGTAACGCCAATAGGGACTTTCCATTGACGTCAATGGGTGGAGTATTTA
CGGTAAACTGCCCACTTGGCAGTACATCAAGTGTATCATATGCCAAGTACGCCCCCTATTGACGTCAATG
ACGGTAAATGGCCCGCCTGGCATTATGCCCAGTACATGACCTTATGGGACTTTCCTACTTGGCAGTACAT
CTACGTATTAGTCATCGCTATTACCATGGTGATGCGGTTTTGGCAGTACATCAATGGGCGTGGATAGCGG
TTTGACTCACGGGGATTTCCAAGTCTCCACCCCATTGACGTCAATGGGAGTTTGTTTTGGCACCAAAATC
AACGGGACTTTCCAAAATGTCGTAACAACTCCGCCCCATTGACGCAAATGGGCGGTAGGCGTGTACGGTG
GGAGGTCTATATAAGCAGAGCTCGTTTAGTGAACCGTCAGATCGCCTGGAGACGCCATCCACGCTGTTTT
GACCTCCATAGAAGACACCGGGACCGATCCAGCCTCCGGACTCTAGAGGATCGAACCCTTGGATCCATGG
GGTGGATTCGTGGTCGGAGGTCCAGGCATTCTTGGGGTATGTTTTTACCACATAAGTGTGTAGTTTTGTT
TTTATACAATTTTAAGATACCAAGAATTTCATACAAGCATATAAGGCATAGGCAAGCAATTTACTCTGAT
TCTTTTCATGTTCTATTTATGTTGATTTCATGTTATGTTCATATTGTATTTTAAATCAAGTCTTCCAAAA
CTTAAAAACTTTTTTCCCTTTATTTTATTAACTATATTATGGTAAAGTCGCTCACAACTTGGGACAGCTT
GAAATAGTGAAATAGATGTGGAGTCAGACTTGAATTTTACTCAAACTTTTACCGCTCTCTAGATTTGAAG
GGTCAATTTGCAACCTCTGTGTAGTTTAGTTTAGTTTCTTTTTCTATCAAATACAGGCATGAAACATTCC
ATATTTATCTTAGATTTGTGTAATATTCAAATCAACTGACATGTATGTTAAAGCACTTTTTATCCTAAAG
GTGTTTATAAATTTATAACTTATTCACAGTTGGTAACATTATATTAAATAAGCTTTATGATTTTGGCCAT
CTCTAGCATTCAATTTCTTCATCC

Table S2 List of primers

Gene	Sequence 5'-3'	Product size
**CRISPR Knock in**		
NKG2D DNA Template	**F-** GACATTTTGAAGTAATTGGCG	549bp
	**R-** CAAGTTGTGAGCGACTTTACC	
**RT-PCR**		
ULBP2-qPCR	**F-** TTCTGTGCCTCCCGCTTCT	100bp
	**R-** AACTTAGGGATGACGGTGATGTC	
CCL3-qPCR	**F-** ACTTGCTGCTGACACGCCGAC	108bp
	**R-** GGCTTGGAGCACTGGCT	
**ChIP-PCR**		
ChIP-ULBP2	**F-** AGGGACGCGATCACAGACA	127bp
	**R-** TACAGGAAAGTGCAGGGAAAAATT	
ChIP-CCL3	**F-** CAGCCCATCAACCCTAT	122bp
	**R-** TTAGTCAGTCCCTTCT	
**ULBP2 construct**		
ULBP2 full CDS-R	CATCCTCCCTGGCATCTGAGGATCC	759
ULBP2 full CDS-F	CTCGAGCCCACCATGGCAGCAGCCGCC	
**ULBP2 Bisulphite pyro-sequencing**		
ULBP2-pyro-F	TGGTTGAGTTTAGAGTATGATAAGTTAGG	179
ULBP2-pyro-R	ATTCCTTCCACCACAAACTCATTTAAT	
ULBP2-pyro-S	AGAGTATGATAAGTTAGGG	
**ULBP2 shRNA sequence**		
TRCN0000056730	CGTGACATTCAGCTGGAGAAT	
TRCN0000056731	CCTCCTCTTTGACTCAGAGAA	

Table S3 List of common upregulated genes

Gene Name	Function	Ref
CACNA1B	CACNA1B is responsible for encoding the pore-forming subunit of the Cav2.2/N-type voltage-gated calcium channel found in pre-synaptic neurons. This channel plays a critical role in facilitating SNARE-mediated neurotransmission, particularly during the early stages of postnatal development.	(1)
CCK	Cholecystokinin (CCK) acts as a key hormonal regulator in the digestive process. CCK cells are primarily concentrated in the proximal small intestine and release the hormone into the bloodstream upon food intake. Its physiological effects include promoting pancreatic secretion and gallbladder contraction, regulating gastric emptying, and eliciting feelings of satiety.	(2)
CCL3	CCL3, a chemokine known for its role in recruiting NK cells and augmenting the production of IFNγ.	(3)
CLDN4	Claudin-4 (CLDN4) is crucial for maintaining epithelial integrity and transport functions. However, in various epithelial cancers like bladder urothelial carcinoma (BUC), colon, gastric, and pancreatic cancers, increased CLDN4 disrupts barriers, preserving the tumor environment, retaining growth factors, and shielding tumors from anticancer drugs. CLDN4 expression correlates with cancer progression in these malignancies.	(4)
CYP1B1	The expression of CYP1B1 serves as a pivotal regulator of redox balance within retinal vascular cells. It holds notable significance in the developmental processes of retinal vasculature and the neovascularization observed in oxygen-induced ischemic retinopathy (OIR).	(5)
HSD11B1	In patients with renal cell carcinoma (RCC), the presence of intratumoral 11 beta-hydroxysteroid dehydrogenase type 1 (HSD11B1), an enzyme that converts inactive glucocorticoids into active forms, was associated with unfavorable clinical outcomes and correlated with immunosuppressive gene signatures. HSD11B1 expression was primarily observed in tumor-infiltrating immune myeloid cells.	(6)
LSMEM2	LSMEM2, a cell surface N-glycoprotein on human cardiomyocytes, remains stable during differentiation and heart development. Its presence at intercalated discs suggests a role in cell junctions or cardiomyocyte coupling.	(7)
MYO7B	Myosin 7a (Myo7a) is vital for the development and function of microvilli and stereocilia. Mutations occurring within the MyTH4-FERM tandems of these myosins have been linked to visual and hearing impairments in affected individuals.	(8)
PACSIN1	PACSIN1 is a peripheral membrane protein, in gliomas, linking its improper expression to neurological disorders. PACSIN1 acts as a prognostic marker factor for brain glioma and gastric cancer.	(9,10)

PCDHGA2	PCDHGA2 is a neural cadherin-like cell adhesion protein that is crucial for forming and maintaining specific cell-cell connections in the brain. PCDHGA2 mutations are frequently observed in Invasive lobular carcinoma (ILC).	(11)
RSPH10B	RSPH10B is likely a component of the axonemal radial spoke complex 3 (RS3), which plays a significant role in ciliary motility.	(12)
TRIM63	TRIM63 is a sensitive and specific biomarker indicative of renal cell carcinoma associated with aberrations in the MiT family.	(13)

References

1. Gorman KM, Meyer E, Grozeva D, Spinelli E, McTague A, Sanchis-Juan A*, et al.* Bi-allelic Loss-of-Function CACNA1B Mutations in Progressive Epilepsy-Dyskinesia. American journal of human genetics **2019**;104(5):948-56.
2. Liddle RA. Cholecystokinin cells. Annual review of physiology **1997**;59:221-42.
3. Allen F, Bobanga ID, Rauhe P, Barkauskas D, Teich N, Tong C*, et al.* CCL3 augments tumor rejection and enhances CD8(+) T cell infiltration through NK and CD103(+) dendritic cell recruitment via IFNgamma. Oncoimmunology **2018**;7(3):e1393598.
4. Maesaka F, Kuwada M, Horii S, Kishi S, Fujiwara-Tani R, Mori S*, et al.* Hypomethylation of CLDN4 Gene Promoter Is Associated with Malignant Phenotype in Urinary Bladder Cancer. International journal of molecular sciences **2022**;23(12).
5. Falero-Perez J, Sorenson CM, Sheibani N. Cyp1b1-deficient retinal astrocytes are more proliferative and migratory and are protected from oxidative stress and inflammation. American journal of physiology Cell physiology **2019**;316(6):C767-C81.
6. Poinot H, Dupuychaffray E, Arnoux G, Alvarez M, Tachet J, Ezzar O*, et al.* Activation of endogenous glucocorticoids by HSD11B1 inhibits the antitumor immune response in renal cancer. Oncoimmunology **2024**;13(1):2286820.
7. Luecke LB, Waas M, Littrell J, Wojtkiewicz M, Castro C, Burkovetskaya M*, et al.* Surfaceome mapping of primary human heart cells with CellSurfer uncovers cardiomyocyte surface protein LSMEM2 and proteome dynamics in failing hearts. Nature cardiovascular research **2023**;2(1):76-95.
8. Li J, He Y, Weck ML, Lu Q, Tyska MJ, Zhang M. Structure of Myo7b/USH1C complex suggests a general PDZ domain binding mode by MyTH4-FERM myosins. Proceedings of the National Academy of Sciences of the United States of America **2017**;114(19):E3776-E85.
9. Liu Z, Li L, Li X, Hua M, Sun H, Zhang S. Prediction and prognostic significance of ALOX12B and PACSIN1 expression in gastric cancer by genome-wide RNA expression and methylation analysis. Journal of gastrointestinal oncology **2021**;12(5):2082-92.
10. Zimu Z, Jia Z, Xian F, Rui M, Yuting R, Yuan W*, et al.* Decreased Expression of PACSIN1 in Brain Glioma Samples Predicts Poor Prognosis. Frontiers in molecular biosciences **2021**;8:696072.
11. Ping Z, Siegal GP, Harada S, Eltoum IE, Youssef M, Shen T*, et al.* ERBB2 mutation is associated with a worse prognosis in patients with CDH1 altered invasive lobular cancer of the breast. Oncotarget **2016**;7(49):80655-63.

12. Konjikusic MJ, Lee C, Yue Y, Shrestha BD, Nguimtsop AM, Horani A, *et al.* Kif9 is an active kinesin motor required for ciliary beating and proximodistal patterning of motile axonemes. Journal of cell science **2023**;136(5).
13. Wang XM, Zhang Y, Mannan R, Skala SL, Rangaswamy R, Chinnaiyan A, *et al.* TRIM63 is a sensitive and specific biomarker for MiT family aberration-associated renal cell carcinoma. Modern pathology : an official journal of the United States and Canadian Academy of Pathology, Inc **2021**;34(8):1596-607.
