# Supplementary material for: Epigenetic modulation elicits an NK cell-mediated immune response in urothelial carcinoma
Source: Mol Med. 2025 Jun 24;31:247. doi: 10.1186/s10020-025-01264-9 (PMC12186328; doi:10.1186/s10020-025-01264-9)
Supplement: Supplementary file 2 — Supplementary Material 2. [file 10020_2025_1264_MOESM2_ESM.pdf]

Ensembl_ID	Symbol	EntrezID	TPM_MB49_25uM_1(B)	TPM_MB49_25uM_2(B)	TPM_MB49_DMSO_48hr_1(A)	TPM_MB49_DMSO_48hr_2(A)	FC B/A 25uM	log2(B/A)	p-value (B/A)	Adj pval
ENSMUSG00000000982	Ccl3	20302	2.942035115	3.563269349	0.77602107	0.755823175	4.246714041	2.086346966	0.015262311	0.222240149
ENSMUSG00000009185	Ccl8	20307	4.74941812	4.006063094	1.163272289	0.849746407	4.349428662	2.120825902	0.014016964	0.214498571
ENSMUSG00000031780	Ccl17	20295	4.721697781	5.642131987	3.180327596	3.942338388	1.455049246	0.541067982	0.113285641	0.437180558
ENSMUSG00000074715	Ccl28	56838	0.10736195	0.070434154	0.092036355	0.119521171	0.840414929	-0.250826305	0.539625963	0.722096102
ENSMUSG00000023235	Ccl25	20300	2.16546673	1.535209113	2.175725427	1.96356668	0.894035924	-0.161595293	0.577325672	0.744820168
ENSMUSG00000035385	Ccl2	20296	1305.332497	1377.923772	575.497021	631.4892663	2.223104187	1.152575562	0.003834276	0.118883645
ENSMUSG00000029379	Cxcl3	330122	488.5424184	383.4163896	217.6865554	207.551896	2.050517316	1.035987927	0.051605702	0.339732516
ENSMUSG00000026166	Ccl20	20297	2.19589564	1.680704631	1.673278366	0.305573831	1.959014562	0.970128122	0.323677217	0.690190914
ENSMUSG00000035373	Ccl7	20306	124.2260412	96.90279854	54.73079938	65.7745446	1.835012726	0.875790069	0.076114913	0.388168833
ENSMUSG00000029380	Cxcl1	14825	194.2486681	171.5923237	130.8543283	108.4586101	1.528713801	0.612318337	0.05793832	0.354887955
ENSMUSG00000035042	Ccl5	20304	1.264037843	2.132394001	1.548000364	0.904625862	1.384814289	0.469692516	0.474585288	0.66527531
ENSMUSG00000018927	Ccl6	20305	2.99385326	1.520592937	1.655799832	1.773973836	1.316251926	0.396435643	0.539392753	0.721912602
ENSMUSG00000019122	Ccl9	20308	21.06433225	18.54377443	19.08234537	21.53276929	0.975206078	-0.036220977	0.801474623	0.897067356
ENSMUSG00000029371	Cxcl5	20311	7.816272654	4.395272659	7.099363987	5.671513726	0.956202509	-0.064611903	0.893910548	0.955547857
ENSMUSG00000034855	Cxcl10	15945	10.62292143	10.68090778	11.68011642	10.54057843	0.95873821	-0.060791163	0.505979406	0.693794135
ENSMUSG00000058427	Cxcl2	20310	77.28828239	71.02480599	83.21475069	79.78905091	0.909875027	-0.136259693	0.175861908	0.53102522
ENSMUSG00000073888	Ccl27a	20301	0.583437144	0.789443299	1.250383129	1.096054894	0.585091287	-0.773266361	0.063335069	0.365498156
