# Supplementary material for: Epigenetic modulation elicits an NK cell-mediated immune response in urothelial carcinoma
Source: Mol Med. 2025 Jun 24;31:247. doi: 10.1186/s10020-025-01264-9 (PMC12186328; doi:10.1186/s10020-025-01264-9)
Supplement: Supplementary file 3 — Supplementary Material 3. [file 10020_2025_1264_MOESM3_ESM.pdf]

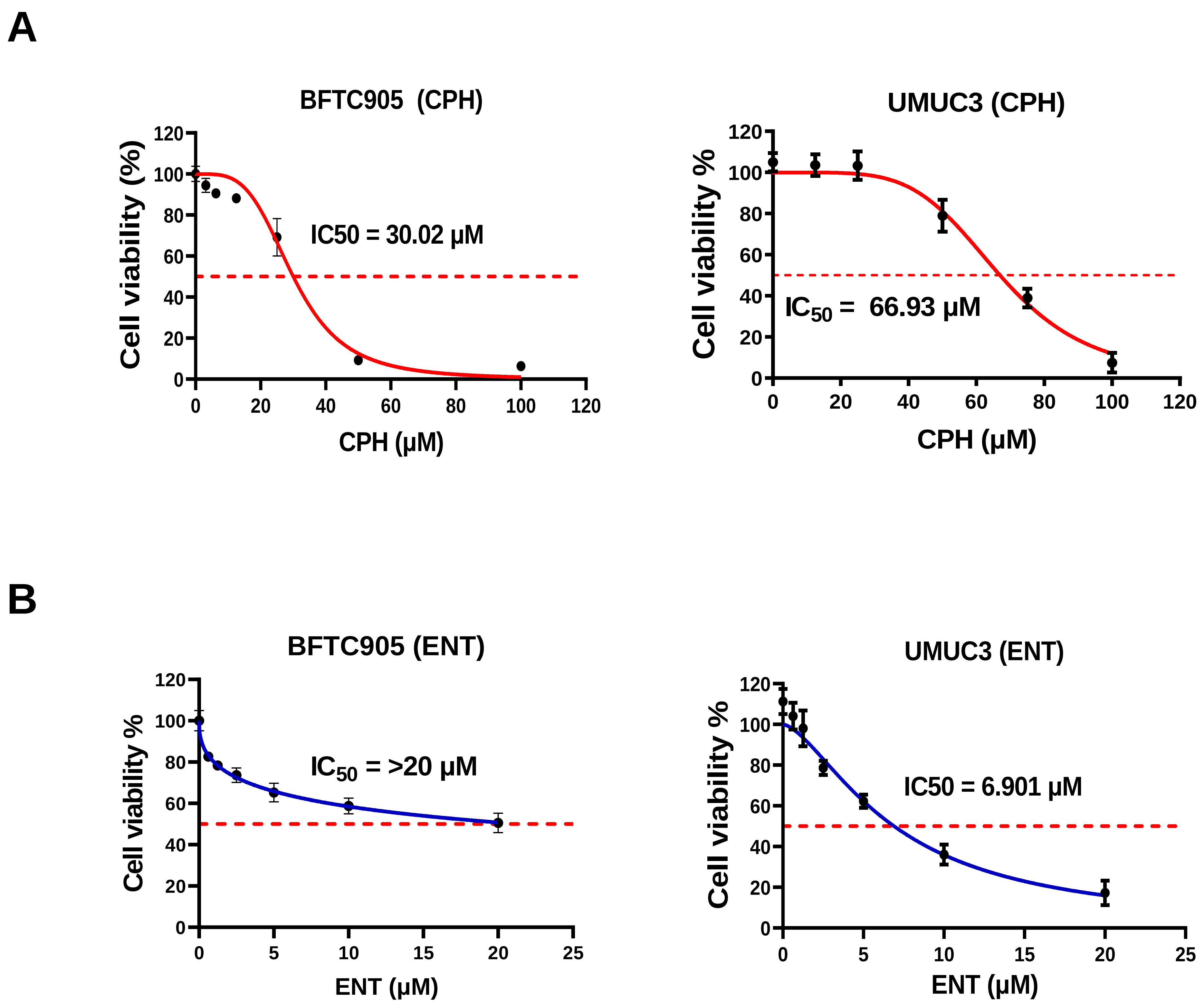

Figure S1. Cytotoxic effect of CPH and ENT on UC cells BFTC905 and UMUC3 treated with (A) CPH or (B) ENT showing cytotoxicity, as analyzed by CCK8 assay. IC50 value is also shown.

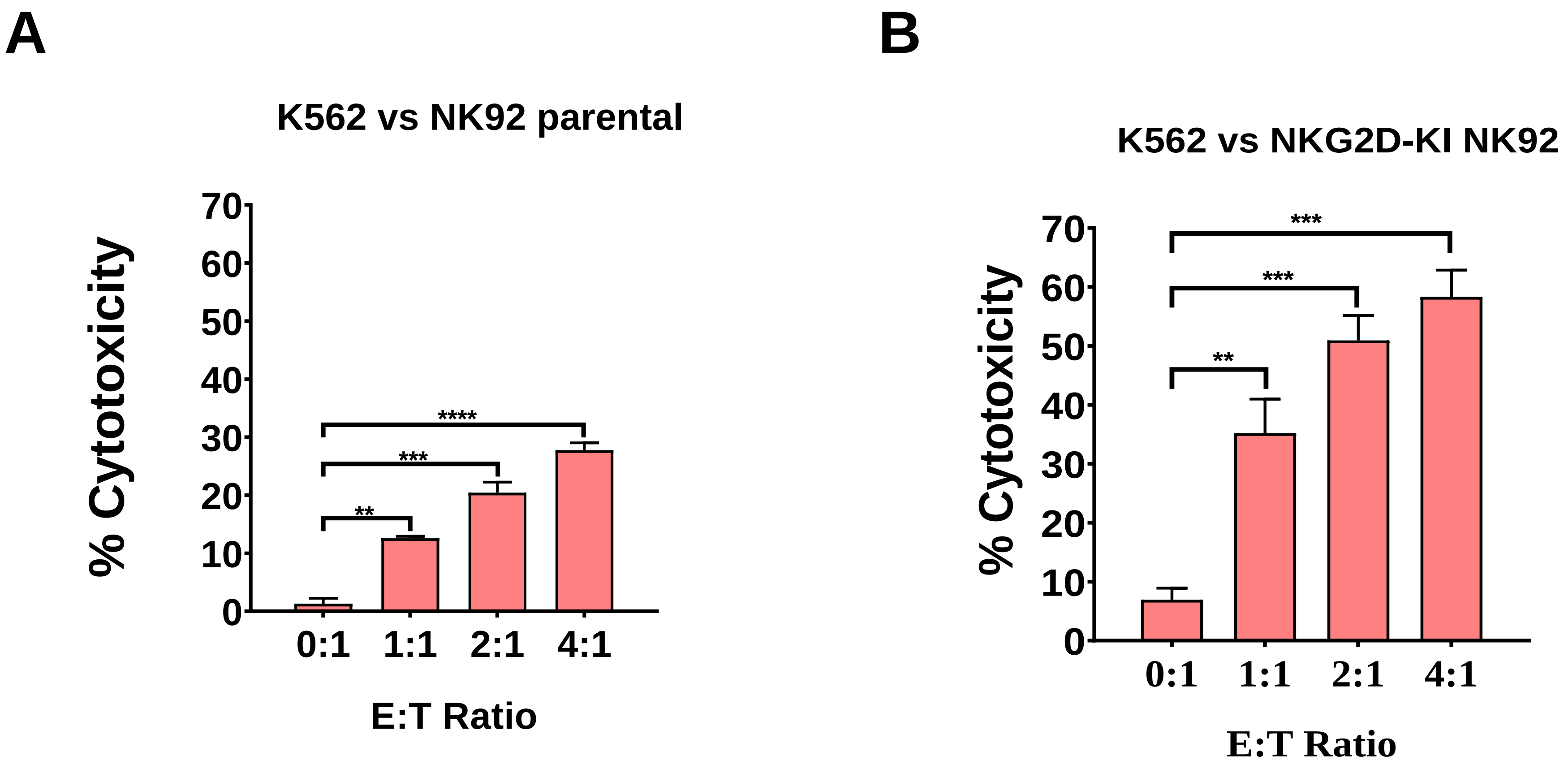

Figure S2. NK mediated cytotoxic assay in K562 cells. (A) Bar chart showing increasing cytotoxicity of K562 cells when co-cultured with (A) NK92 parental and (B) NKG2D-NK92 cells. Cell specific lysis as the measure of cytotoxicity is quantified using Calcein-AM assay. Each error bar represents mean ± SD from triplicates. The significance is calculated by unpaired T-test. ****P<0.0001; *** P<0.0005; **P<0.01; *P<0.05.

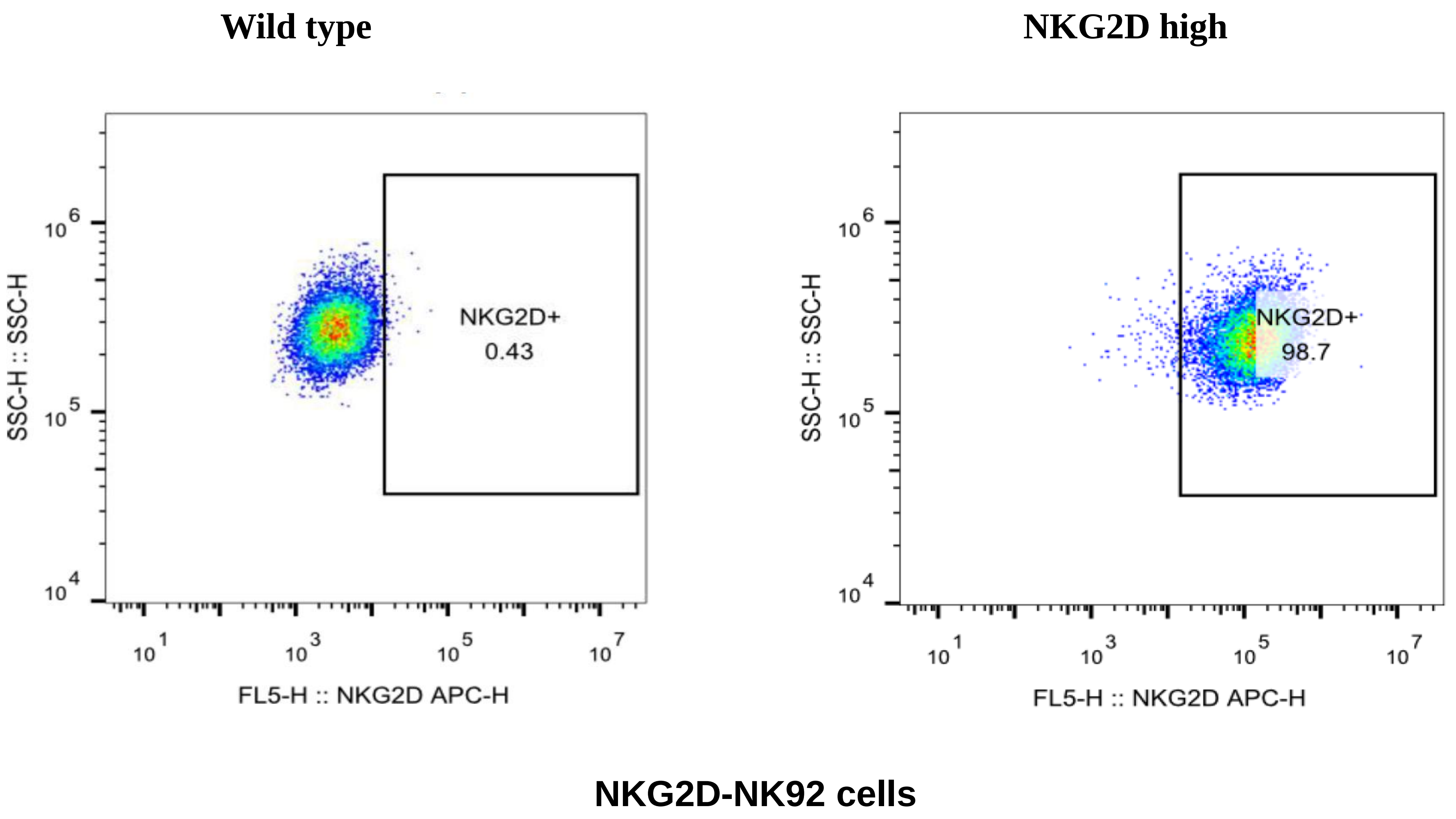

Figure S3. Expression of NKG2D in NK92 cells. Flow cytometry analysis of NKG2D level in (left panel) NK92 cells and (right panel) NKG2D knock-in NK92 cells.

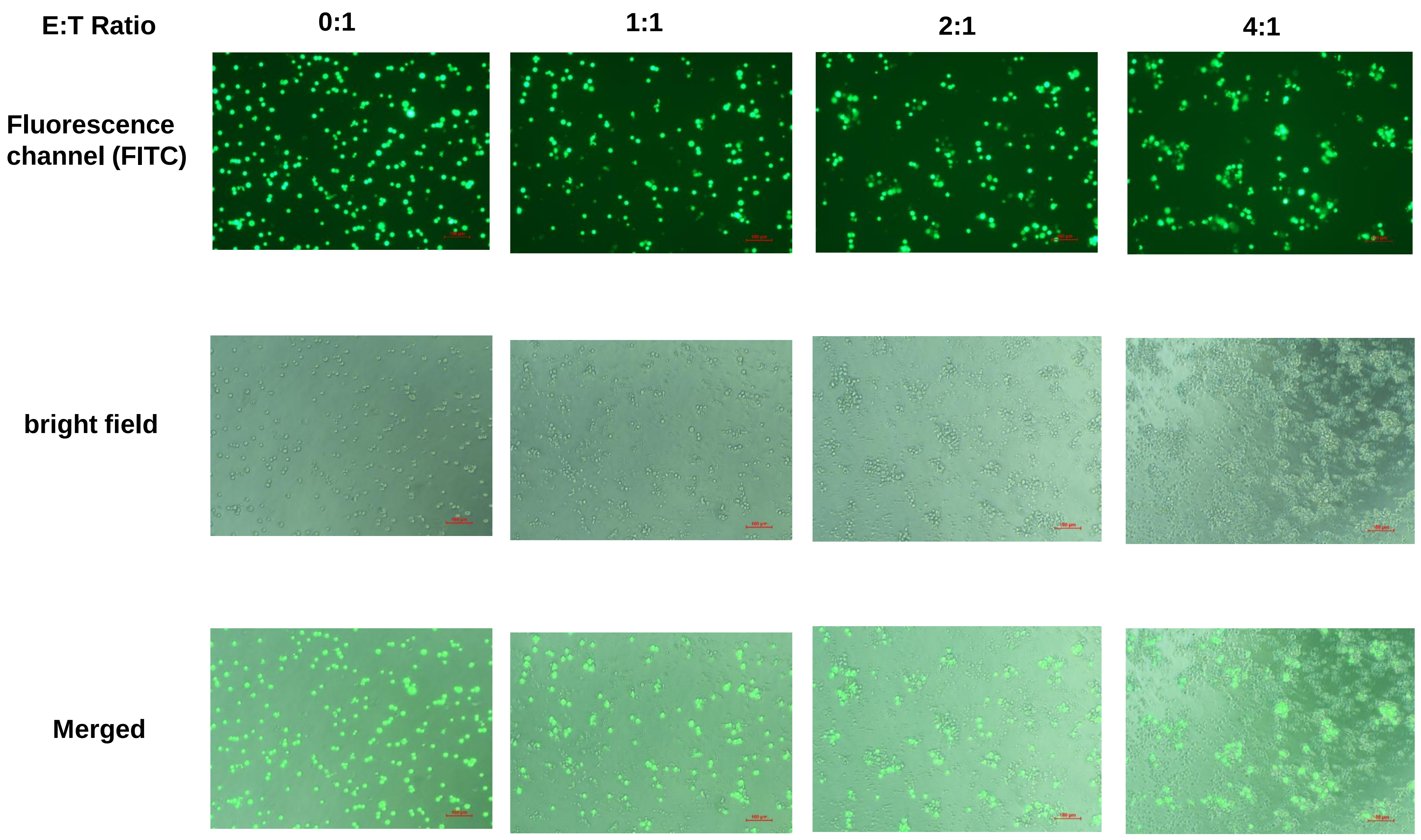

Figure S4. Representative fluorescent microscopic images showing co-culture of NKG2D-NK92 cells with Calcein-AM labeled BFTC905 UC cells pre-treated with CPH. Top panel: fluorescent channel (FITC) showing Calcein-AM +ve cancer cells; middle panel: bright field showing both NK and UC cells; bottom panel: merged images.

Human NK cell purity

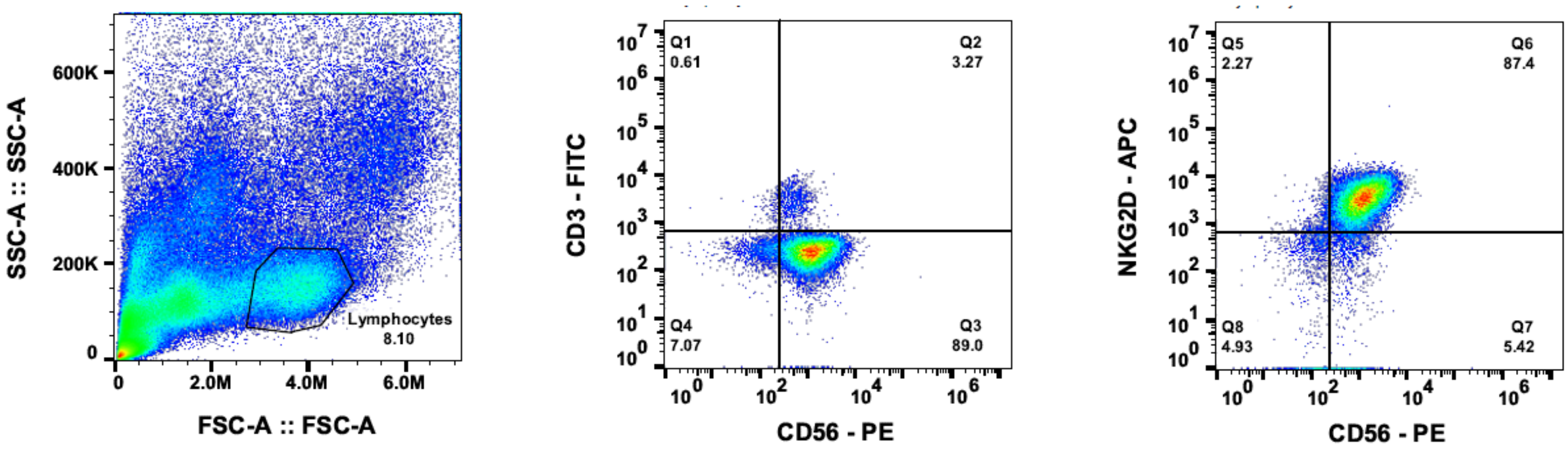

Figure S5. Representative Flow cytometry analysis for purity of human primary NK cells: **Left panel:** Scatter plot showing FSC vs SSC gated population of PBMC from cord blood cells. **Middle panel:** Scatter plot showing 89% of CD3-ve and CD56+ve population of the gated cells. **Right panel:** Scatter plot showing NKG2D and CD56 double positive human primary NK cells as 87.4% of the gated cells .

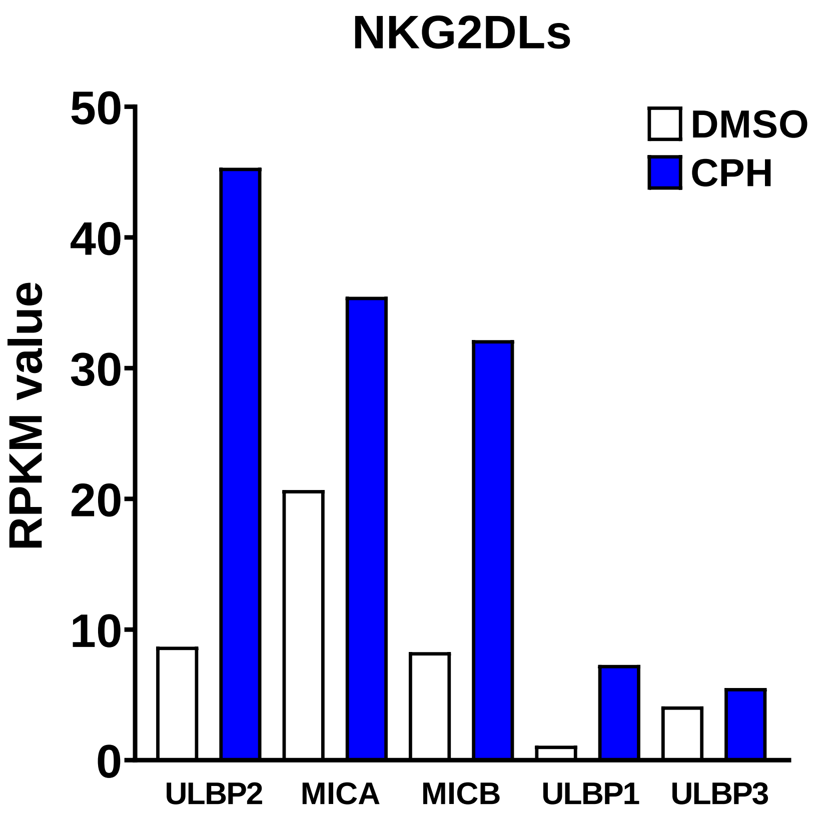

Figure S6. mRNA expression of various NKG2DLs after CPH treatment. Bar chart showing the expression (RPKM value) of NKG2DLs in BFTC905 cells treated with 55µM CPH for 24 hrs based on our previously published RNA-Seq data. Data referenced from Jou et al., 2021 (Reference 33)

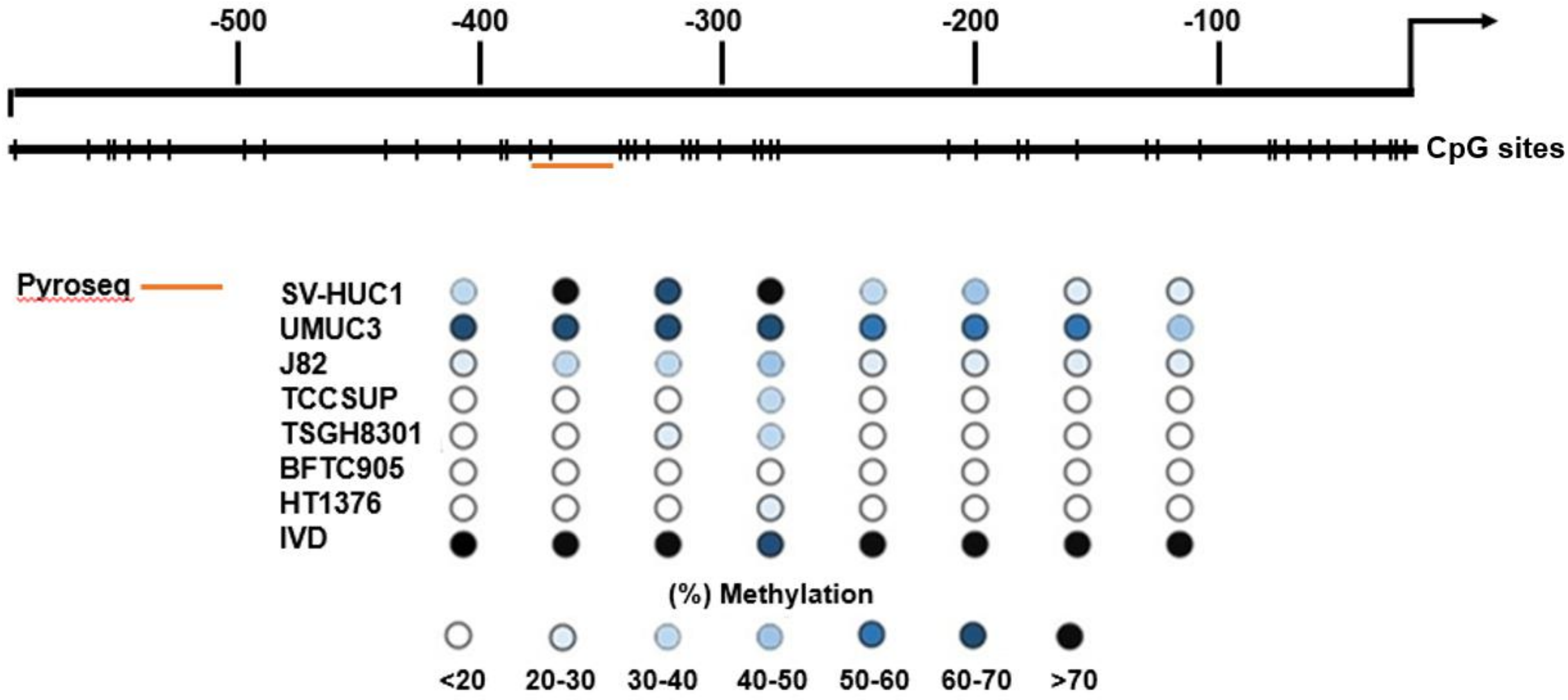

Figure S7. Methylation percentage in UC cells by pyrosequencing. Top panel: diagram showing the location of the CpG sites in the promoter region of ULBP2. Region for bisulfite pyrosequencing is shown in orange line. Lower panel: Figure showing the methylation percentage of CpG sites in UC cells in promoter region of ULBP2 via bisulphite pyrosequencing.

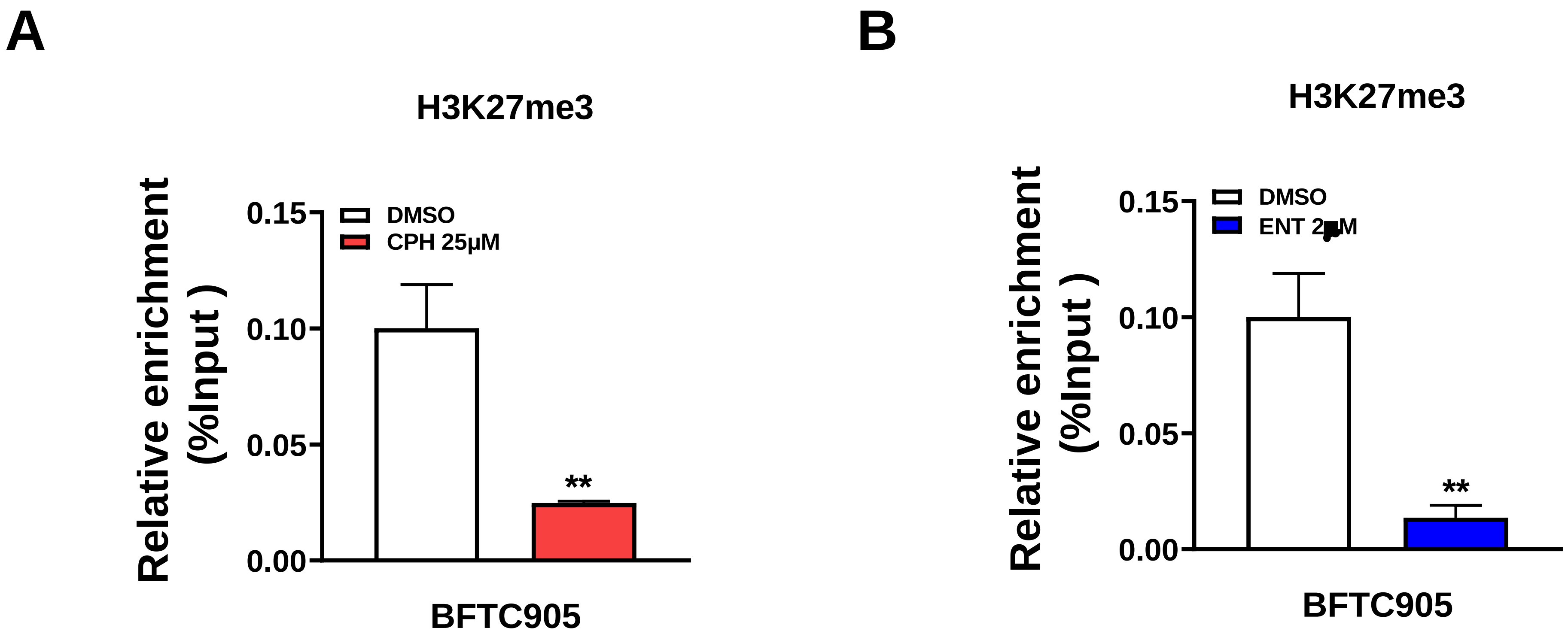

Figure S8. Impact of CPH on repressive mark H3K27me3 in ULBP2 promoter. BFTC905 cells illustrating reduced enrichment of repressive mark H3K27me3, Bar chart showing significant reduced enrichment (A) and (B) in the promoter region of ULBP2 analyzed by ChIP qPCR. Each error bar represents mean ± SD from triplicates. The significance is calculated by unpaired T-test. **$P<0.01$

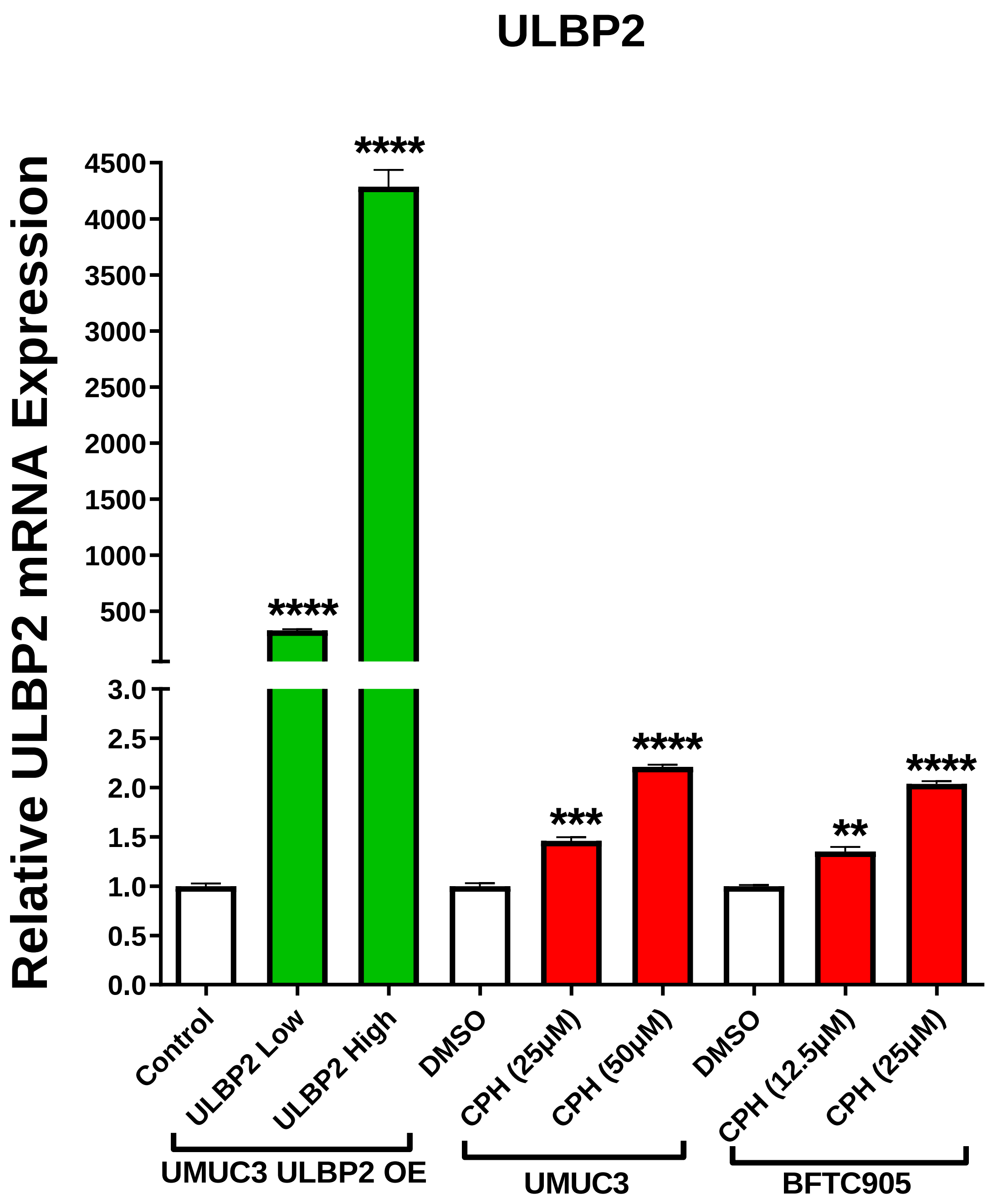

Figure S9. Expression of ULBP2 in ULBP2 overexpressing and CPH-treated UC cells. Relative expression level of ULBP2 in control or ULBP2-overexpressed UMUC3 cells (low: ULBP2-L, high: GFP-H) and UC (UMUC3 and BFTC905) cells treated with various concentration of CPH was determined by quantitative RT-PCR. Bar chart showing relative mRNA expression of ULBP2. Each error bar represents mean ± SD from triplicates. The significance is calculated by unpaired T-test. . *** P<0.0005; **P<0.01; *P<0.05

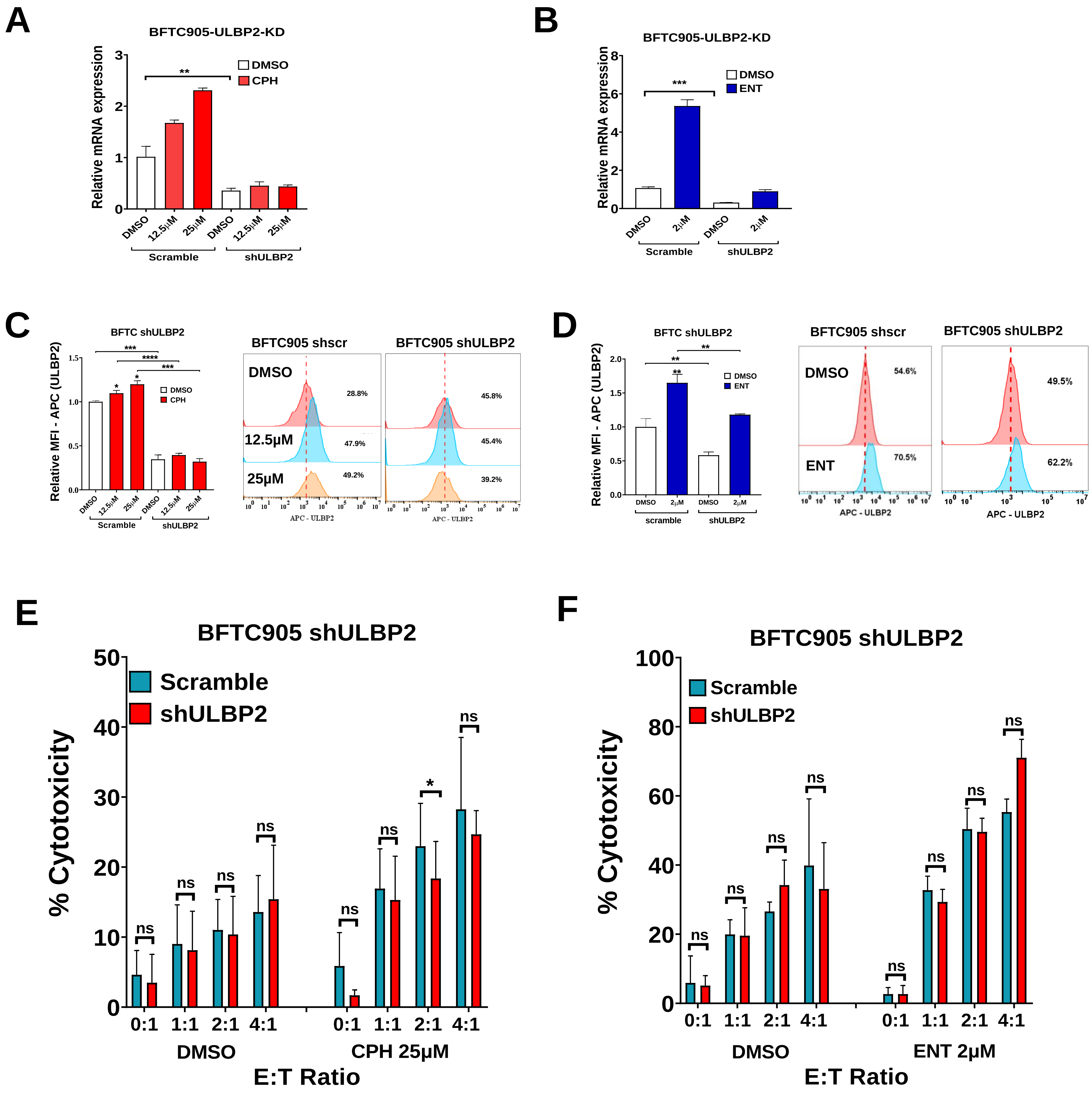

Figure S10. Depletion of ULBP2 in BFTC905 UC cells. BFTC905 cells transiently infected with lentiviral expression shRNA against ULBP2. Relative expression level of ULBP2 in control or ULBP2 depleted cells treated with (A) CPH or (B) ENT was determined by quantitative RT-PCR and Flow cytometry FACS analysis (C) CPH or (D) ENT. Bar chart showing cell specific lysis via Calcein-AM assay with different E:T ratio of NKG2D-NK92 cells vs control or ULBP2 depleted BFTC905 cells pretreated with (D) CPH or (E) ENT. Each error bar represents mean ± SD from triplicates. The significance is calculated by unpaired T-test. *** P<0.0005; **P<0.01; *P<0.05.

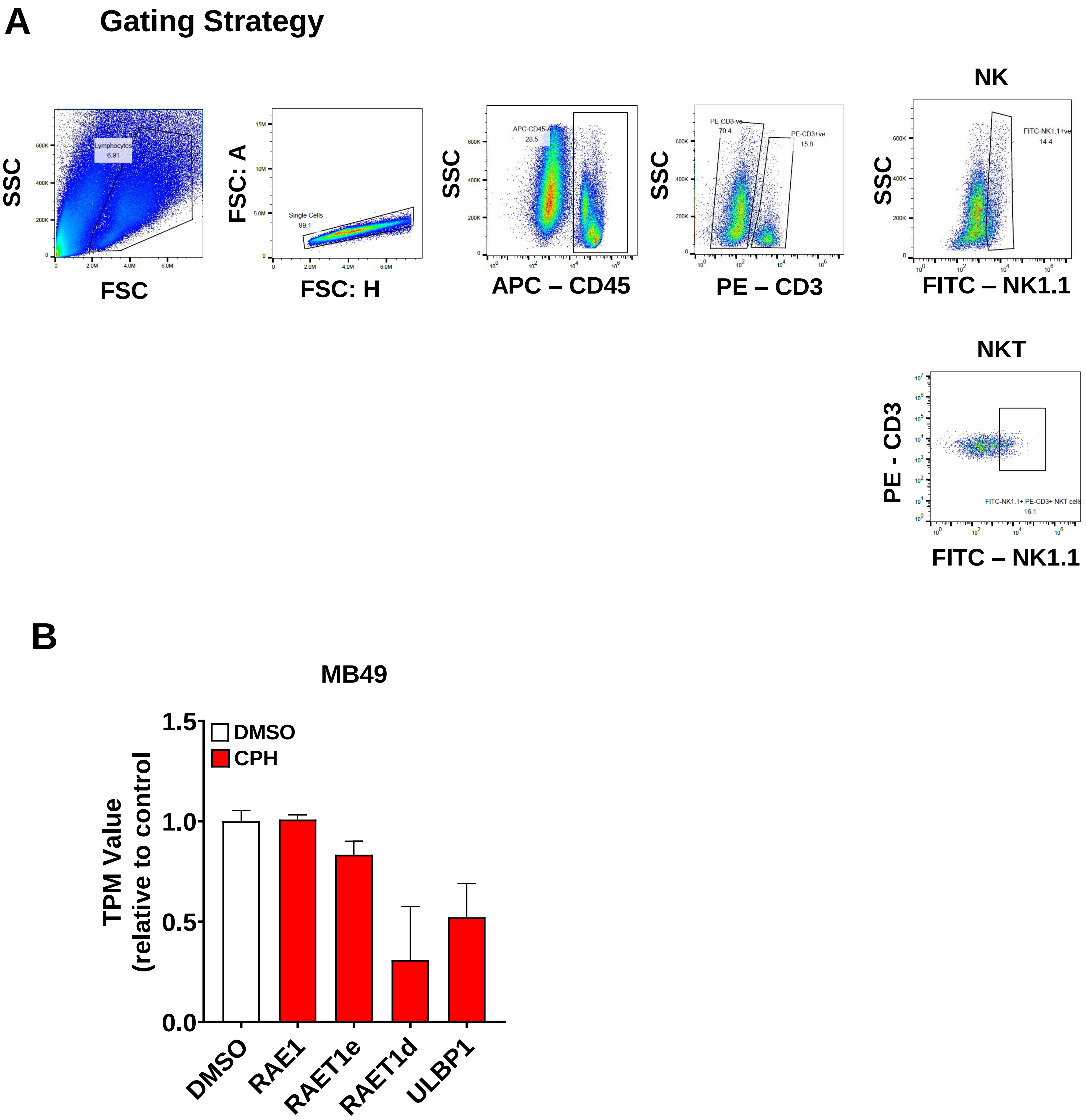

Figure S11. Flow cytometry analysis of TILs in MB49 tumors. (A) Gating strategy for investigating infiltrated NK and NKT cells in MB49 tumor in syngeneic mice model. The scatter plot shows CD45+ve gated population which is further divided into $CD3^{-ve}$ $NK1.1^{+ve}$ NK cells and $CD3^{+ve}$ $NK1.1^{+ve}$ NKT cells (B) mRNA expression level of NKG2DLs in MB49 cells treated with CPH, as determined by RNA-Seq.
